# Comparison of Multidimensional Frailty Instruments for Estimation of Long-term Patient-Centered Outcomes After Cardiac Surgery

**DOI:** 10.1001/jamanetworkopen.2022.30959

**Published:** 2022-09-09

**Authors:** Louise Y. Sun, Habib Jabagi, Jiming Fang, Douglas S. Lee

**Affiliations:** 1 Division of Cardiac Anesthesiology, University of Ottawa Heart Institute, Ottawa, Ontario, Canada; 2 Cardiovascular Research Program, Institute for Clinical Evaluative Sciences, Toronto, Ontario, Canada; 3 School of Epidemiology and Public Health, University of Ottawa, Ottawa, Ontario, Canada; 4 Division of Cardiac Surgery, Valley Health System, Ridgewood, New Jersey; 5 Division of Cardiology, University Health Network and Peter Munk Cardiac Centre, Toronto, Ontario, Canada

## Abstract

**Importance:**

Little is known about the performance of available frailty instruments in estimating patient-relevant outcomes after cardiac surgery.

**Objective:**

To examine how well the Johns Hopkins Adjusted Clinical Groups (ACG) frailty indicator, the Hospital Frailty Risk Score (HFRS), and the Preoperative Frailty Index (PFI) estimate long-term patient-centered outcomes after cardiac surgery.

**Design, Setting, and Participants:**

This retrospective cohort study was conducted in Ontario, Canada, among residents 18 years and older who underwent coronary artery bypass grafting or aortic, mitral or tricuspid valve, or thoracic aorta surgery between October 2008 and March 2017. Long-term care residents, those with discordant surgical encounters, and those receiving dialysis or dependent on a ventilator within 90 days were excluded. Statistical analysis was conducted from July 2021 to January 2022.

**Main Outcomes and Measures:**

The primary outcome was patient-defined adverse cardiovascular and noncardiovascular events (PACE), defined as the composite of severe stroke, heart failure, long-term care admission, new-onset dialysis, and ventilator dependence. Secondary outcomes included mortality and individual PACE events. The association between frailty and PACE was examined using cause-specific hazard models with death as a competing risk, and the association between frailty and death was examined using Cox models. Areas under the receiver operating characteristic curve (AUROC) were determined over 10 years of follow-up for each frailty instrument.

**Results:**

Of 88 456 patients (22 924 [25.9%] female; mean [SD] age, 66.3 [11.1] years), 14 935 (16.9%) were frail according to ACG criteria, 63 095 (71.3%) according to HFRS, and 76 754 (86.8%) according to PFI. Patients with frailty were more likely to be older, female, and rural residents; to have lower income and multimorbidity; and to undergo urgent surgery. Patients meeting ACG criteria (hazard ratio [HR], 1.66; 95% CI, 1.60-1.71) and those with higher HFRS scores (HR per 1.0-point increment, 1.10; 95% CI, 1.09-1.10) and PFI scores (HR per 0.1-point increment, 1.75; 95% CI, 1.73-1.78) had higher rates of PACE. Similar magnitudes of association were observed for each frailty instrument with death and individual PACE components. The HFRS had the highest AUROC for estimating PACE during the first 2 years and death during the first 4 years, after which the PFI had the highest AUROC.

**Conclusions and Relevance:**

These findings could help to tailor the use of frailty instruments by outcome and follow-up duration, thus optimizing preoperative risk stratification, patient-centered decision-making, candidate selection for prehabilitation, and personalized monitoring and health resource planning in patients undergoing cardiac surgery.

## Key Points

**Question:**

How do various multidimensional frailty instruments perform in estimating patient-centered outcomes after cardiac surgery?

**Findings:**

In this cohort study that included 88 456 patients who underwent cardiac surgery, the Hospital Frailty Risk Score had the highest area under the receiver operating characteristic curve (AUROC) for estimating patient-defined cardiovascular and noncardiovascular events during the first 2 postoperative years and death during the first 4 years, after which the Perioperative Frailty Index had the highest AUROC.

**Meaning:**

The varying performance of multidimensional frailty instruments in patients undergoing cardiac surgery in this study suggests that the use of these instruments could be tailored to optimize preoperative risk stratification, patient-centered decision-making, candidate selection for prehabilitation, and personalized postoperative and long-term monitoring and health resource planning.

## Teaser

This cohort study examines the performance of the Johns Hopkins Adjusted Clinical Groups (ACG) frailty indicator, the Hospital Frailty Risk Score, and the Preoperative Frailty Index.

## Introduction

Over the last century, life expectancy around the world has more than doubled from an average of 29 years in 1900 to 73 years in 2022.^1^ This remarkable achievement is attributed to improved nutrition, hygiene,^2^ and, importantly, advances in medical and surgical management of cardiovascular disease.^3^ Accordingly, modern patients with cardiac concerns more often present with advanced comorbidities and physical frailty, posing significant challenges for clinicians in the perioperative setting.^4^ Frailty is prevalent in approximately 20% of patients undergoing cardiac surgery^4,5^ and is characterized by diminished physiologic reserve and limited ability to tolerate or maintain homeostasis in response to surgical stress.^5^ Frailty effectively increases patients’ susceptibility to procedure failure, adverse outcomes, disability, and death irrespective of comorbidities and chronological age.^4,5,6,7,8^

To date, much cardiovascular research has focused on clinician-centric outcomes, such as death and major adverse cardiovascular events (MACE). However, a national consensus-based study of patients with cardiovascular disease, caregivers, and clinicians found that the ability to live independently^9,10,11^ and being free from severe stroke, heart failure (HF), dialysis, and ventilator assistance were more important from the patient’s perspective. This composite outcome, termed patient-defined adverse cardiovascular and noncardiovascular events (PACE),^12^ adds a valuable perspective for surgical decision-making, especially among patients with frailty.

Despite its prognostic importance, frailty instruments are heterogeneous^9,10^ and thus not a part of routine cardiac surgery risk stratification.^13,14^ The advent of big data readily enables recalibration of existing risk tools by incorporating frailty as an important metric. Of the available frailty instruments, the Johns Hopkins Adjusted Clinical Groups (ACG) frailty indicator, the Hospital Frailty Risk Score (HFRS), and the Preoperative Frailty Index (PFI) are multidimensional instruments most suited for routinely collected data. Although these instruments have limitations compared with original measures (eg, the Frailty Index), particularly for having rudimentary information on functional performance, they capture elements of frailty that denote susceptibility to adverse outcomes in situations of stress, such as surgery. The comparative strengths of these frailty instruments in the context of patient-oriented cardiovascular surgical outcomes, such as PACE, has not, to our knowledge, been evaluated. To inform future outcome-based research, we conducted a population-based study in Ontario, Canada, to compare the performance of these frailty instruments in estimating patient-relevant outcomes over varying durations of follow-up after cardiac surgery.

## Methods

The data set from this study is held securely in coded form at ICES. The use of data was authorized under section 45 of Ontario’s Personal Health Information Protection Act, which does not require review by a research ethics board or informed consent.^5^ Our reporting followed the Strengthening the Reporting of Observational Studies in Epidemiology (STROBE) guideline.^15^

### Design and Population

We conducted a population-based, retrospective cohort study of adult patients aged 20 to 105 years who underwent index coronary artery bypass grafting or aortic, mitral or tricuspid valve, or thoracic aorta surgery in Ontario between October 1, 2008, and March 31, 2017. We excluded non-Ontario residents, those with discordant surgical encounters, those receiving dialysis or with ventilator dependence within 90 days, and those already residing in long-term care (LTC) facilities immediately prior to surgery (eFigure 1 in the Supplement). Ontario is the most populous province in Canada, with a publicly funded health care system serving 14.6 million ethnically diverse residents.^16^

### Data Sources

We used the CorHealth Ontario clinical registry and ICES population-level administrative health care databases with information from all Ontario residents. Data sets were linked deterministically using confidential identifiers. CorHealth maintains a prospective registry of all patients who undergo invasive cardiac procedures in Ontario and regularly undergoes selected chart audits and core laboratory validation.^17^

We linked the CorHealth registry (patient and procedural details) with Canadian Institute for Health Information Discharge Abstract Database (DAD; comorbidities, hospital admissions, and in-hospital procedures) and Same Day Surgery database (SDS; comorbidities), Assistive Devices Program (ADP; ventilator use), National Rehabilitation Reporting Service (NRS; inpatient rehabilitation), Continuing Care Reporting System (CCRS; long-term care admissions), the Canadian Organ Replacement Registry (CORR; dialysis), the Ontario Health Insurance Plan database (OHIP; physician service claims), and the Registered Persons Database (RPDB; vital statistics). These databases have been validated for many outcomes, exposures, and comorbidities, including HF, chronic obstructive pulmonary disease, asthma, hypertension, and diabetes.^18,19,20,21,22,23,24,25^

### Baseline Patient Characteristics

Patient characteristics were identified from the CorHealth registry and supplemented with data from DAD, SDS, and OHIP, using codes from *International Statistical Classification of Diseases and Related Health Problems Tenth Revision, Canada* (ICD-10-CA)^22^ within 5 years prior to the index surgical procedure according to validated algorithms.^18,20,23,24,25^ We estimated each patient’s socioeconomic status by using the neighborhood median income from the Canadian census,^26^ and determined residence status (rural vs urban) using Statistics Canada definitions.^27^ Height, weight, and body mass index (BMI; calculated as weight in kilograms divided by height in meters squared) were identified from the CorHealth Ontario registry and used to determine extreme or class III obesity (defined as weight >159 kg or BMI ≥40).^28,29,30,31^

### Exposure

Frailty may be assessed based on clinical^32^ and/or functional criteria^33,34^ or based a constellation of frailty-defining diagnoses.^4,7,35^ The latter provides multidimensional assessment and is adaptable to routinely collected data.^36^ Of these, the Johns Hopkins ACG frailty indicator, HFRS, and PFI have been validated and applied with success in patients undergoing surgery.

The ACG frailty indicator is designed and validated for use with administrative data and has been used to assess outcomes and resource utilization in Ontario surgical patients.^4,7,37,38^ It is a binary classifier based on 12 clusters of frailty-defining diagnoses (eTable 1A in the Supplement), where patients meeting criteria for at least 1 diagnostic cluster are designated as being frail.^4,39^ Due to its proprietary nature, we are unable to provide specific diagnostic codes used to define this indicator.

The HFRS is derived and validated using routinely collected hospitalization data of patients aged 75 years or older from the United Kingdom.^40,41^ This score comprises 109 differentially weighted *ICD-10* codes (eTable 1B in the Supplement) and ranges between 0 and 173.1 points.^40^ We assessed the HFRS both as a continuous variable and with it categorized in quartiles.

The PFI is an accumulating deficits index (eTable 1C in the Supplement) modeled after the original Canadian Study of Health and Aging Frailty Index.^9,42,43,44^ It is calculated by dividing the sum of the scores representing the severity of each deficit by the total number of deficits. The PFI has been validated in patients undergoing noncardiac surgery and is robust in the presence of missing data and variable substitution.^44^ In addition, both PFI^44^ and HFRS^45,46^ have been previously shown to be associated with 1-year mortality in the noncardiac surgical setting. We assessed the PFI as a continuous variable as well as having it categorized into quintiles.

### Outcomes

The primary outcome was PACE, defined as the composite of severe stroke necessitating hospitalization for at least 14 days or inpatient rehabilitation, new onset dialysis, HF, LTC admission, or ventilator dependence.^12^ Secondary outcomes were all-cause mortality and individual components of PACE. As with our previous research,^47^ severe stroke was identified using the DAD^48^ and NRS, ventilator dependence using the ADS and OHIP, HF using a validated algorithm with 85% sensitivity and 97% specificity,^25^ LTC admission using the CCRS, and dialysis using the DAD, SDS, OHIP, and CORR. Mortality was ascertained using the RPDB, and hospitalization duration using the DAD.

### Statistical Analysis

We compared continuous variables with a 2-sample *t* test or Wilcoxon rank sum test where appropriate and categorical variables with a χ^2^ test. Events were assessed from the date of the procedure until March 31, 2020. Patients were censored when they lost OHIP eligibility.

To account for death as a competing event, we estimated the cumulative incidence of PACE using cumulative incidence functions, compared event rates between groups using the Fine and Gray test of inequality, and assessed the association between frailty and the rate of PACE using cause-specific hazard models. Survival probabilities in the groups with and without frailty were calculated using the Kaplan-Meier method, mortality rates were compared using the log-rank test, and the association between frailty and death was assessed using Cox proportional hazard models. We plotted receiver operating characteristic (ROC) curves for each frailty instrument, and areas under the ROC (AUROC) as a function of time. We used 0.5 to 0.6 (failed), 0.6 to 0.7 (poor), 0.7 to 0.8 (fair), 0.8 to 0.9 (good), and 0.9 to 1.0 (excellent) to interpret AUROCs.^49,50^ To avoid redundant adjustment of risk factors that may already be a part of the frailty instruments, we did not control for medical comorbidities.

We repeated our time-to-event modeling while sequentially adjusting for (1) demographic variables and (2) the combination of demographic characteristics and comorbidities listed in the Table. As with our previous studies,^5,51^ demographic variables included age, sex, rurality, socioeconomic status, and urgent procedure performed during acute inpatient admissions.

**Table.** Baseline Characteristics by ACG Frailty Status

Variable	Patient, No. (%)			*P* value
	ACG nonfrail (n = 73 521)	ACG frail (n = 14 935)	Total (N = 88 456)	
Female	18 127 (24.7)	4797 (32.1)	22 924 (25.9)	<.001
Male	55 394 (75.3)	10 138 (67.9)	65 532 (74.1)	
Age, mean (SD), y	65.7 (11.1)	69.2 (10. 9)	66.3 (11.1)	<.001
Rural residence	11 237 (15.3)	2404 (16.1)	13 641 (15.4)	.01
Income quintile				
1, Lowest	13 504 (18.4)	3343 (22.4)	16 847 (19.0)	<.001
2	15 052 (20.5)	3291 (22.0)	18 343 (20.7)	
3	15 077 (20.5)	2908 (19.5)	17 985 (20.3)	
4	14 987 (20.4)	2726 (18.3)	17 713 (20.0)	
5, Highest	14 901 (20.3)	2667 (17.9)	17 568 (19.9)	
Remote MI	12 389 (16.9)	3282 (22.0)	15 671 (17.7)	<.001
Recent MI	16 796 (22.8)	5524 (37.0)	22 320 (25.2)	<.001
History of PCI	7609 (10.3)	1694 (11.3)	9303 (10.5)	<.001
Hypertension	59 872 (81.4)	13 087 (87.6)	72 959 (82.5)	<.001
Atrial fibrillation	8921 (12.1)	2782 (18.6)	11 703 (13.2)	<.001
LVEF				
≥50%	49 241 (67.0)	8957 (60.0)	58 198 (65.8)	<.001
35%-49%	14 570 (19.8)	3387 (22.7)	17 957 (20.3)	
20%-34%	5909 (8.0)	1571 (10.5)	7480 (8.5)	
<20%	1159 (1.6)	355 (2.4)	1514 (1.7)	
Unknown	2642 (3.6)	665 (4.5)	3307 (3.7)	
Heart failure	19 114 (26.0)	5860 (39.2)	24 974 (28.2)	<.001
Cerebrovascular disease	6013 (8.2)	2094 (14.0)	8107 (9.2)	<.001
Peripheral arterial disease	6584 (9.0)	2020 (13.5)	8604 (9.7)	<.001
COPD or asthma	13 464 (18.3)	3829 (25.6)	17 293 (19.5)	<.001
Diabetes	29 179 (39.7)	7251 (48.6)	36 430 (41.2)	<.001
Morbid obesity	24 020 (32.7)	4746 (31.8)	28 766 (32.5)	.03
Hypothyroidism	910 (1.2)	393 (2.6)	1303 (1.5)	<.001
Anemia	1775 (2.4)	747 (5.0)	2522 (2.9)	<.001
Dialysis	1009 (1.4)	425 (2.8)	1434 (1.6)	<.001
Kidney disease	3830 (5.2)	1485 (9.9)	5315 (6.0)	<.001
Liver disease	991 (1.3)	348 (2.3)	1339 (1.5)	<.001
Primary malignant neoplasm	3362 (4.6)	886 (5.9)	4248 (4.8)	<.001
Metastatic malignant neoplasms	356 (0.5)	100 (0.7)	456 (0.5)	.004
Dementia	1-5^a^	345-349^a^	350 (0.4)	<.001
Urgent surgery	3231 (4.4)	711 (4.8)	3942 (4.5)	.048
HFRS categories				
0	24 861 (33.8)	500 (3.3)	25 361 (28.7)	<.001
0.1-2.0	19 817 (27.0)	1949 (13.0)	21 766 (24.6)	
2.1-5.0	17 111 (23.3)	4725 (31.6)	21 836 (24.7)	
≥5.1	11 732 (16.0)	7761 (52.0)	19 493 (22.0)	
HFRS, mean (SD)	2.5 (3.3)	7.0 (5.6)	3.3 (4.2)	<.0001
PFI categories				
0.00-0.04	10 956 (14.9)	746 (5.0)	11 702 (13.2)	<.001
0.05-0.10	21 947 (29.9)	2737 (18.3)	24 684 (27.9)	
0.11-0.20	27 582 (37.5)	5529 (37.0)	33 111 (37.4)	
0.21-0.44	13 016 (17.7)	5843 (39.1)	18 859 (21.3)	
0.45-1.00	20 (0.0)	80 (0.5)	100 (0.1)	
PFI, mean (SD)	0.12 (0.08)	0.18 (0.09)	0.13 (0.09)	<.001
Death	15 735 (21.4)	5472 (36.6)	21 207 (24.0)	<.001
PACE	15 766 (21.4)	4621 (30.9)	20 387 (23.0)	<.001
Stroke	1533 (2.1)	422 (2.8)	1955 (2.2)	<.001
Heart failure	10 664 (14.5)	2389 (16.0)	13 053 (14.8)	<.001
Long-term care	2436 (3.3)	1443 (9.7)	3879 (4.4)	<.001
Dialysis	3330 (4.5)	1178 (7.9)	4508 (5.1)	<.001
Ventilator dependence	122 (0.2)	46 (0.3)	168 (0.2)	<.001

Abbreviations: ACG, Johns Hopkins Adjusted Clinical Groups; COPD, chronic obstructive pulmonary disease; HFRS, Hospital Frailty Risk Score; LVEF, left ventricular ejection fraction; MI, myocardial infarction; PACE, patient-defined adverse cardiovascular and noncardiovascular events; PCI, percutaneous coronary intervention; PFI, Preoperative Frailty Index.

^a^ Small cells containing 5 or fewer patients are suppressed to prevent the risk of reidentification, per ICES privacy policy.

We performed the analysis using SAS version 9.4 (SAS Institute). Statistical significance was defined as a 2-sided *P* < .05.

## Results

### Prevalence of Frailty

A total of 88 456 patients were included in this study (22 924 [25.9%] women; mean [SD] age, 66.3 [11.1] years). The median (IQR) follow-up duration was 6.2 (4.1-8.5) years, and the maximum follow-up was 11.5 years. Frailty was present in 14 935 patients (16.9%) when assessed by the ACG indicator. When assessed by the HFRS and PFI, 53 095 (71.3%) and 76 754 (86.8%) exhibited some degree of frailty as evidenced by nonzero scores. Frail patients tended to be older and female, irrespective of the instrument used (Table) and were more likely to be rural residents, have lower income status, have lower left ventricular ejection fraction and lower BMI, have higher comorbidity burden, and undergo urgent surgery. Baseline characteristics by frailty instrument are provided in eTable 2 in the Supplement. eTable 3 in the Supplement summarizes the sensitivity, specificity, and positive and negative predictive values of each instrument.

### Outcomes

#### PACE

**Figure 1.**
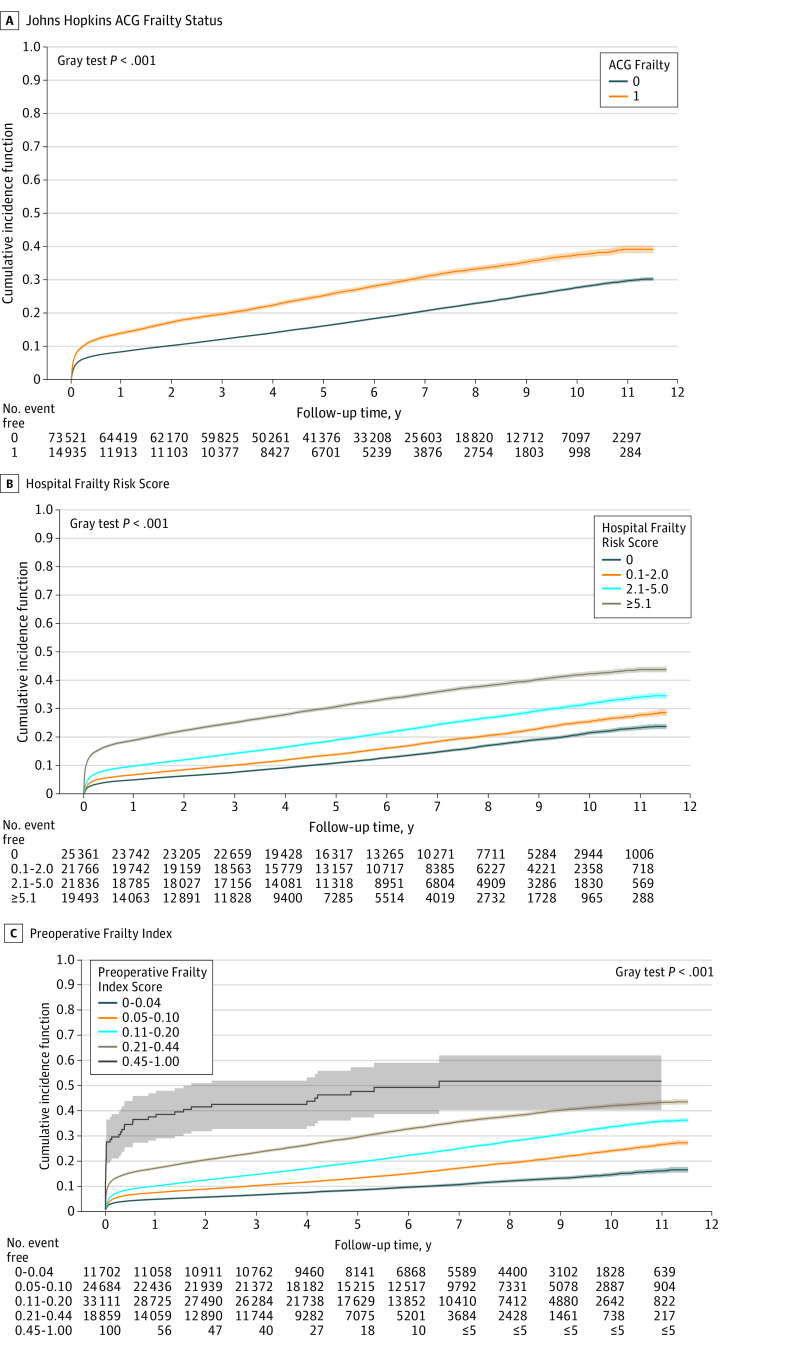
Unadjusted Cumulative Incidence of Patient-Defined Adverse Cardiovascular and Noncardiovascular Events After Cardiac Surgery, by Frailty Instrument The shaded areas represent 95% confidence intervals. ACG indicates Johns Hopkins Adjusted Clinical Groups.

**Figure 2.**
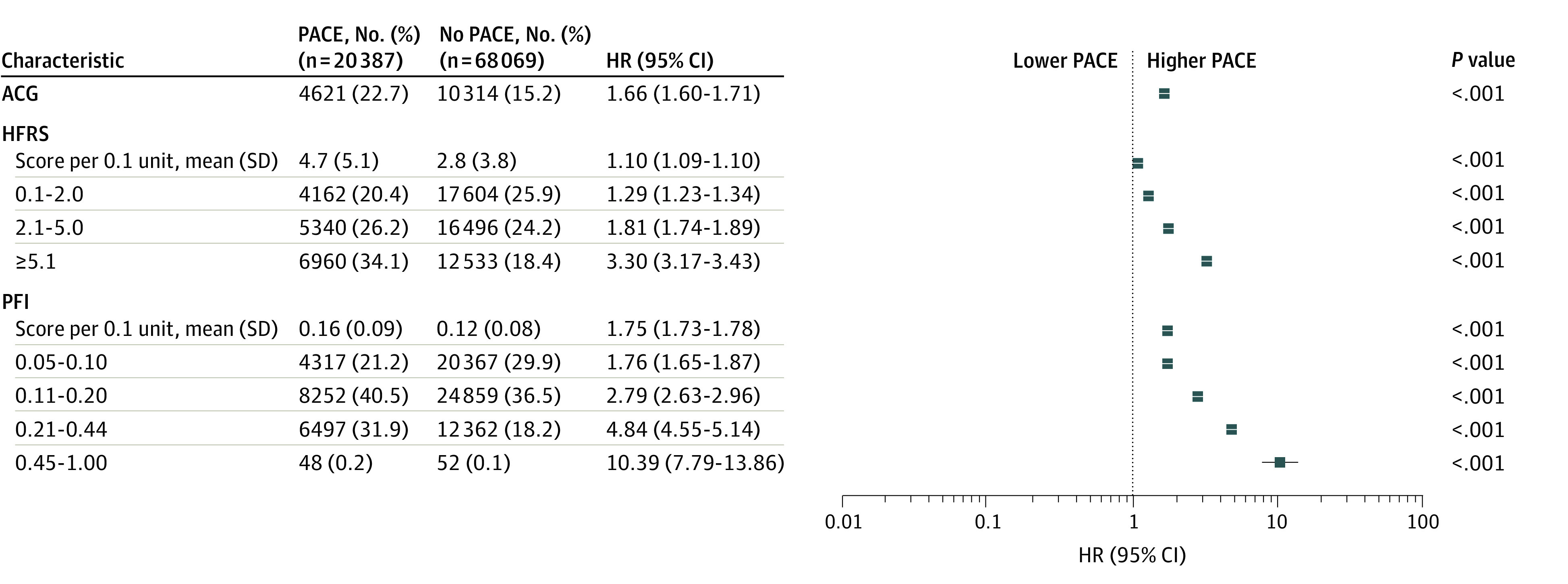
Unadjusted Cumulative Incidence and Hazard Ratios (HRs) of Patient-Defined Adverse Cardiovascular and Noncardiovascular Events (PACE) for Each Frailty Instrument ACG indicates Johns Hopkins Adjusted Clinical Groups; HFRS, Hospital Frailty Risk Score; and PFI, Preoperative Frailty Index.

PACE occurred in 20 387 patients (23.0%). Figure 1 illustrates the cumulative incidence of PACE according to each frailty instrument, and Figure 2 the unadjusted association between frailty and PACE. PACE was more frequent in older (mean [SD] age, 69.4 [10.7] years vs 65.3 [11.1] years; *P* < .001), female (6258 [27.3%] vs 14 129 [21.6%]; *P* < .001), and frail individuals (Figure 2). Of those meeting ACG frailty criteria, 4621 (22.7%) developed PACE vs 10 314 (15.2%) in those who were not frail. The HFRS and PFI scores were higher in those who developed PACE vs those who did not (mean HFRS: 4.7 [5.1] vs 2.8 [3.8]; *P* < .001; mean PFI: 0.16 [0.09] vs 0.12 [0.08]; *P* < .001).

Patients meeting ACG frailty criteria (unadjusted hazard ratio [HR], 1.66; 95% CI, 1.60-1.71) and those with higher HFRS scores (unadjusted HR per 1.0-point increment, 1.10; 95% CI, 1.09-1.10) and PFI scores (unadjusted HR per 0.1-point increment, 1.75; 95% CI, 1.73-1.78) had higher rates of PACE (Figure 2) and individual PACE events (eFigure 2A in the Supplement).

**Figure 3.**
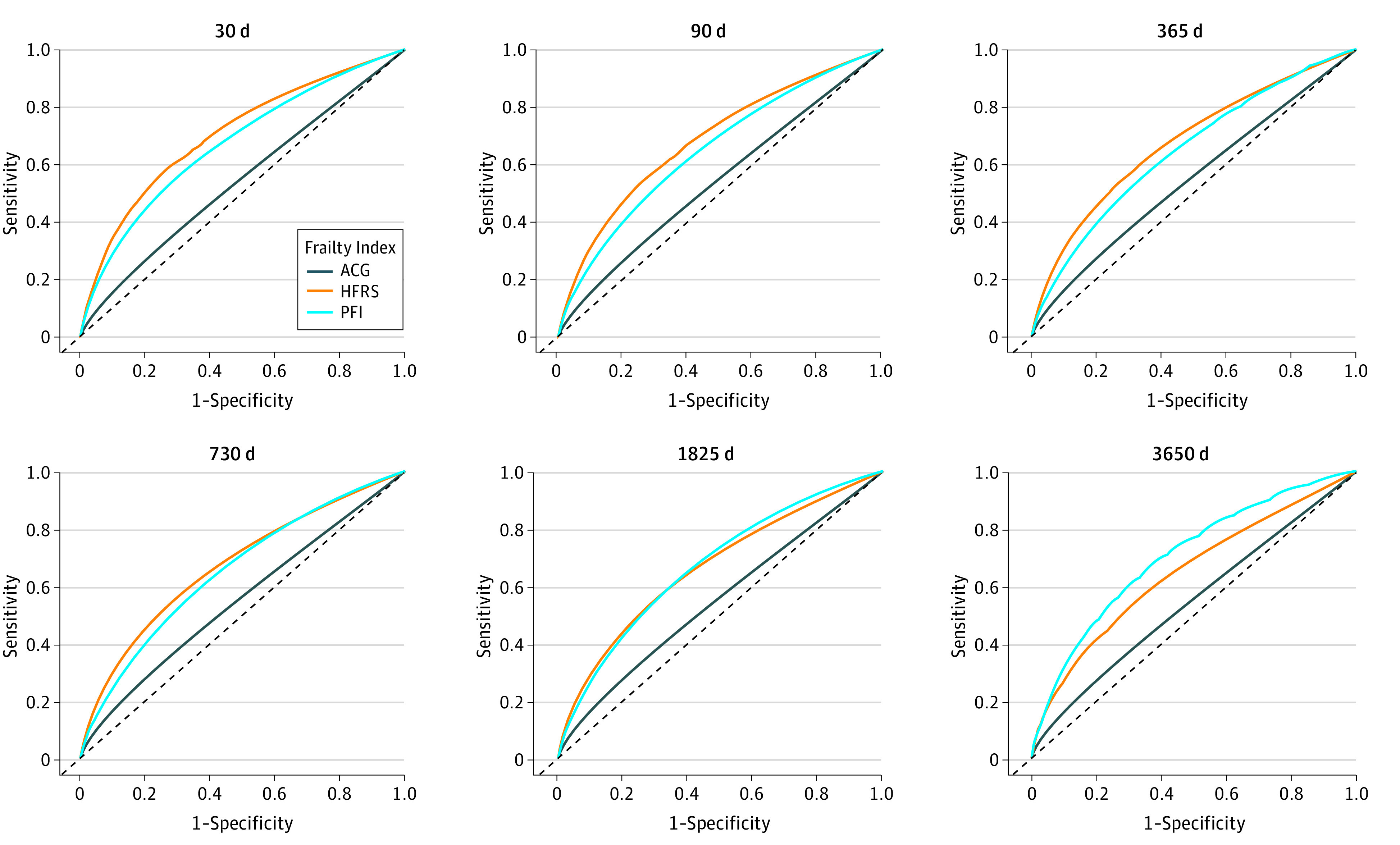
Unadjusted Time-Dependent Receiver Operating Characteristic Curves for the Estimation of Patient-Defined Adverse Cardiovascular and Noncardiovascular Events ACG indicates Johns Hopkins Adjusted Clinical Groups; HFRS, Hospital Frailty Risk Score; and PFI, Preoperative Frailty Index.

**Figure 4.**
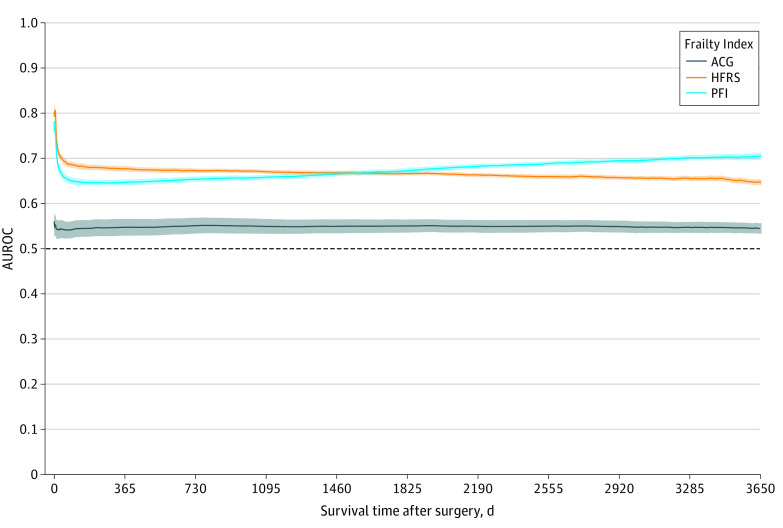
Plot of Unadjusted Areas Under the Receiver Operating Characteristic Curves (AUROCs) Over Time for the Estimation of Patient-Defined Adverse Cardiovascular and Noncardiovascular Events The shaded areas represent 95% CIs. ACG indicates Johns Hopkins Adjusted Clinical Groups; HFRS, Hospital Frailty Risk Score; and PFI, Preoperative Frailty Index.

The time-dependent ROC curves and corresponding AUROCs as a function of time are presented in Figures 3 and 4, respectively. The differences in AUROC between instruments are presented in eTable 4 in the Supplement. The HFRS had the highest AUROC for estimating PACE in the first 4 postoperative years, after which the AUROC of PFI was highest. Conversely, the ACG indicator had the lowest AUROC throughout the follow-up period. While none of the instruments estimated HF well (time-averaged AUROC 0.52-0.57), the HRFS was good in estimating dialysis (time-averaged AUROC, 0.79; 95% CI 0.78-0.80) and fair in estimating LTC admission (AUROC, 0.72; 95% CI, 0.71-0.73), while the PFI was fair both in the estimation of dialysis (AUROC, 0.76; 95% CI, 0.76-0.77) and LTC (AUROC, 0.73; 95% CI, 0.72-0.74). The AUROCs for all individual PACE events are provided in eFigure 2B in the Supplement.

#### Death

Death occurred in 21 207 patients (24.0%) and was more frequent in older (mean [SD] age, 71.6 [10.4] years vs 64.6 [10.9] years; *P* < .001), female (6540 [28.5%] vs 14 667 [22.4%]; *P* < .001), and frail individuals. Of those meeting ACG criteria, 5472 (25.8%) died vs 9463 (14.1%) of those who were not frail. The HFRS and PFI scores were higher in those who died vs those who survived (mean [SD] HFRS score: 5.5 [5.5] vs 2.6 [3.4]; *P* < .001; mean [SD] PFI score: 0.19 [0.09] vs 0.12 [0.08]; *P* < .001).

Long-term survival by frailty instrument is illustrated in eFigure 3 in the Supplement, and the HRs of the association between frailty and death are demonstrated in eFigure 4 in the Supplement. Patients meeting ACG frailty criteria (unadjusted HR, 1.92; 95% CI, 1.86-1.98), and those with higher HFRS scores (unadjusted HR per 1.0-point increment, 1.11; 95% CI, 1.11-1.12) and PFI scores (unadjusted HR per 0.1-point increment, 2.23; 95% CI, 2.20-2.26) had higher rates of death.

The time-dependent ROC curves and corresponding AUROCs for death as a function of time are presented in eFigures 5 and 6 in the Supplement, respectively. The differences in AUROC between frailty instruments are presented in eTable 4 in the Supplement. The HFRS had the highest AUROC for predicting death in the first 2 years, after which the AUROC of PFI became highest. Conversely, the ACG indicator had the lowest AUROC the follow-up period.

### Sensitivity Analysis

Our findings remained robust after sequentially adjusting for demographic variables and the combination of demographics and comorbidities (eTables 5 and 6 and eFigures 3 and 7 in the Supplement).

## Discussion

To our knowledge, this is the largest study to date to examine the long-term outcomes in patients with frailty undergoing cardiac surgery from a patient-centered perspective. The novelty of our study lies in its patient-oriented approach and the long duration of follow-up. Four main findings are derived from this study. First, the prevalence of frailty and associated long-term rates of PACE and death are high after cardiac surgery. Second, frailty is most likely to be identified by the PFI and least likely by the ACG frailty indicator. Third, the HFRS had the highest AUROC for estimating PACE and death in the short to medium term; the PFI had the highest AUROC thereafter. Fourth, the AUROC of the HRFS was excellent for estimating new-onset dialysis and moderate for estimating LTC admission. Our findings suggest that the selection of frailty instrument could be tailored to specific outcomes and follow-up durations to better inform patient-centered decision-making, preoperative optimization, and health resource planning. Frailty is associated with operative and long-term mortality,^4,8,52^ cardiac resuscitation,^37,53^ failure to rescue,^52,54^ prolonged hospital and intensive care unit stay,^4,53^ surgical site infection,^4^ nonhome discharge,^52,54^ and readmission,^37^ all of which contribute to poor quality of life and increased health care resource utilization.

### Mortality

Previous studies reported higher rates of perioperative and short-term mortality in patients with frailty after cardiac surgery.^8,55,56^ Lee et al^8^ reported odds ratios of 1.8 (95% CI, 1.1-3.0) for in-hospital mortality and 1.5 (95% CI, 1.1-2.2) at 2 years in association with frailty after cardiac surgery. We found a larger magnitude of long-term association between frailty and mortality. Our findings are directionally concordant with that reported in other cardiac surgery studies.^4,5^

### PACE

An innovative aspect of our study is the use of patient-defined outcomes. Cardiovascular research has long been dominated by so-called tombstone outcomes of death and MACE, whereas PACE was developed through a patient-led process, with input from experienced caregivers and clinicians.^12^ The concept of patient-defined outcomes is especially relevant in this very high-risk population.^12,29^

### Comparing Frailty Instruments

Wide ranges of prevalence and frailty-related mortality have been reported in the cardiac surgery literature, depending on the instrument used.^4^ Previous comparative research has also focused on single-dimensional frailty instruments and were limited to small sample sizes, with few studies reaching beyond 2 years of follow-up.^9,46,52,57,58,59^

Nonetheless, several studies have demonstrated limited agreement between instruments in the estimation of all-cause mortality in medical patients,^36^ while surgical studies mainly used frailty instruments in the functional and physical domain and are generalizable only when data are collected prospectively in select groups of patients undergoing noncardiac surgery. In a bayesian comparison of the Risk Analysis Index–Administrative (RAI-A) and 5-item modified Frailty Index (mFI-5) in 50 630 patients undergoing noncardiac surgery,^60^ the RAI-A performed better than mFI-5 in estimating death and postoperative complications. In a prospective cohort of 645 patients undergoing elective noncardiac surgery, the Clinical Frailty Scale (CFS) performed better than the Fried Phenotype (FP) and PFI in estimating a variety of postoperative outcomes.^61^ Furthermore, when a comparison was made between the Study of Osteoporotic Fractures (SOF) and FP instruments in 167 patients undergoing elective cardiac surgery, patients categorized as frail by SOF were significantly more likely to experience postoperative complications, and poor agreement was found between instruments (FP frail: 47 patients; SOF frail: 15 patients; κ = 0.185).^62^

In a systematic review that compared 9 frailty instruments across 8 cardiac surgery studies, multidimensional instruments outperformed single dimensional instruments in estimating mortality and MACE up to 6 months after surgery.^11^ This possibly explains the higher performance of PFI and HFRS observed in our study relative to the dichotomic ACG indicator.

We observed variable ability across instruments in the estimation of individual PACE events. While none of the instruments estimated HF well (AUROCs, 0.52-0.57), the AUROC for HFRS was good for predicting dialysis (0.79) and for LTC admission (0.72). This information is particularly relevant for optimizing the estimation of specific patient-centered events.

### Performance of Frailty Instruments Over Time

The consistent poor long-term performance of the ACG indicator was likely attributed to its dichotomic nature. The HFRS outperformed the PFI for the estimation of death up to 2 years after cardiac surgery and PACE up to 4 years, after which the PFI performed better. The better long-term performance of the PFI over HFRS is likely attributed to its emphasis on chronic conditions as well as social determinants of long-term health. This time-varying pattern could be used to tailor the choice of frailty instrument according to desired follow-up duration. Specifically, the HFRS may be better suited in the perioperative and acute care setting, whereas the PFI may more appropriately be applied in the estimation of long-term disability and chronic care resource consumption.

### Frailty and Preoperative Risk Stratification

Despite its prognostic importance,^34,63,64,65^ frailty is not a part of the commonly used Society of Thoracic Surgeons mortality score^13^ or the EuroSCORE II.^14^ This is an important knowledge gap and represents missed opportunities to enable evidence-based selection of candidates for physical and nutritional prehabilitation and patient-centered allocation of resources, such as postoperative telemonitoring to mitigate complications.

### Limitations

Our study has several limitations. First, our comparisons are limited to administrative frailty instruments. Although these instruments have limitations relative to physical measures of frailty, they still denote vulnerability in situations of stress and could readily enable population-based studies to inform policy. Second, we were unable to assess the physical aspects of frailty. Third, cohort studies are by nature subject to residual confounding. Nonetheless, our study is, to or knowledge, the largest comparative study in patients undergoing cardiac surgery and explored patient-defined outcomes over up to 10 years of follow-up.

## Conclusions

In this study, frailty was highly prevalent in patients undergoing cardiac surgery, and the performance of frailty instruments varied according to specific outcomes and follow-up durations. Our findings provide new insight for patient-centered surgical decision-making, candidate selection for prehabilitation, and personalized postoperative monitoring and long-term resource planning.

## Notes

Supplement. **eTable 1.** Frailty Measures **eTable 2.** Baseline Characteristics of Participants by Frailty Instrument **eTable 3.** Sensitivity, Specificity, Positive and Negative Predictive Values of Each Frailty Instrument for the Estimation of PACE and Death **eTable 4.** Differences in AUROC Between Frailty Instruments **eTable 5.** Adjusted Hazard Ratios of PACE by Frailty Instrument **eTable 6.** Adjusted Hazard Ratios of Death by Frailty Instrument **eFigure 1.** Patient Flow Diagram **eFigure 2.** Unadjusted Hazard Ratios and Unadjusted Receiver Operating Characteristic Curves for Individual PACE Events and Each Frailty Instrument **eFigure 3. **Time-Dependent Receiver Operating Characteristic Curves of the Frailty Instruments for the Estimation of PACE **eFigure 4. **Unadjusted Estimated Long-term Survival by Each Frailty Index **eFigure 5.** Unadjusted Hazard Ratios of the Association Between Death and Each Frailty Instrument **eFigure 6. **Unadjusted Time-Dependent Receiver Operating Characteristic Curves and Plots of Areas Under the Receiver Operating Characteristic Curve (AUROC) of the Frailty Instruments for the Estimation of Death **eFigure 7.** Time-Dependent Receiver Operating Characteristic Curves of the Frailty Instruments for the Estimation of Death
