# Supplementary material for: Comparison of Multidimensional Frailty Instruments for Estimation of Long-term Patient-Centered Outcomes After Cardiac Surgery
Source: JAMA Netw Open. 2022 Sep 9;5(9):e2230959. doi: 10.1001/jamanetworkopen.2022.30959 (PMC9463609; doi:10.1001/jamanetworkopen.2022.30959)
Supplement: Supplement. — eTable 1. Frailty Measures eTable 2. Baseline Characteristics of Participants by Frailty Instrument eTable 3. Sensitivity, Specificity, Positive and Negative Predictive Values of Each Frailty Instrument for the Estimation of PACE and Death eTable 4. Differences in AUROC Between Frailty Instruments eTable 5. Adjusted Hazard Ratios of PACE by Frailty Instrument eTable 6. Adjusted Hazard Ratios of Death by Frailty Instrument eFigure 1. Patient Flow Diagram eFigure 2. Unadjusted Hazard Ratios and Unadjusted Receiver Operating Characteristic Curves for Individual PACE Events and Each Frailty Instrument eFigure 3. Time-Dependent Receiver Operating Characteristic Curves of the Frailty Instruments for the Estimation of PACE eFigure 4. Unadjusted Estimated Long-term Survival by Each Frailty Index eFigure 5. Unadjusted Hazard Ratios of the Association Between Death and Each Frailty Instrument eFigure 6. Unadjusted Time-Dependent Receiver Operating Characteristic Curves and Plots of Areas Under the Receiver Operating Characteristic Curve (AUROC) of the Frailty Instruments for the Estimation of Death eFigure 7. Time-Dependent Receiver Operating Characteristic Curves of the Frailty Instruments for the Estimation of Death [file jamanetwopen-e2230959-s001.pdf]

Supplemental Online Content

Sun LY, Jabagi H, Fang J, Lee DS. Comparison of multidimensional frailty instruments for estimation of long-term patient-centered outcomes after cardiac surgery. *JAMA Netw Open.* 2022;5(9):e2230959. doi:10.1001/jamanetworkopen.2022.30959

eTable 1. Frailty Measures

eTable 2. Baseline Characteristics of Participants by Frailty Instrument

eTable 3. Sensitivity, Specificity, Positive and Negative Predictive Values of Each Frailty Instrument for the Estimation of PACE and Death

eTable 4. Differences in AUROC Between Frailty Instruments

eTable 5. Adjusted Hazard Ratios of PACE by Frailty Instrument

eTable 6. Adjusted Hazard Ratios of Death by Frailty Instrument

eFigure 1. Patient Flow Diagram

eFigure 2. Unadjusted Hazard Ratios and Unadjusted Receiver Operating Characteristic Curves for Individual PACE Events and Each Frailty Instrument

eFigure 3. Time-Dependent Receiver Operating Characteristic Curves of the Frailty Instruments for the Estimation of PACE

eFigure 4. Unadjusted Estimated Long-term Survival by Each Frailty Index

eFigure 5. Unadjusted Hazard Ratios of the Association Between Death and Each Frailty Instrument

eFigure 6. Unadjusted Time-Dependent Receiver Operating Characteristic Curves and Plots of Areas Under the Receiver Operating Characteristic Curve (AUROC) of the Frailty Instruments for the Estimation of Death

eFigure 7. Time-Dependent Receiver Operating Characteristic Curves of the Frailty Instruments for the Estimation of Death

This supplemental material has been provided by the authors to give readers additional information about their work.

© 2022 Sun LY et al. *JAMA Network Open.*

eTable 1. Frailty Measures

eTable 1a. Johns Hopkins ACG frailty-defining diagnosis indicator[61]

Domains	Examples
Malnutrition	Nutritional Marasmus<br>Other severe protein-calorie malnutrition
Dementia	Senile dementia with delusional or depressive features<br>Senile dementia with delirium
Impaired Vision	Profound impairment, both eyes<br>Moderate or severe impairment, better eye/ lesser eye: profound s
Decubitus Ulcer	Decubitus ulcer
Incontinence of Urine	Incontinence without sensory awareness<br>Continuous leakage
Loss of Weight	Abnormal loss of weight and underweight<br>Feeding difficulties and mismanagement t
Incontinence of Feces	Incontinence of feces
Obesity (morbid)	Morbid obesity
Poverty	Lack of housing<br>And adequate housing<br>Inadequate material resources<br>g
Barriers to Access to Care	No medical facility for care
Difficulty in Walking	Difficulty walking<br>Abnormality of gait
Fall	Fall on stairs or steps<br>Fall from wheelchair

© 2022 Sun LY et al. *JAMA Network Open.*

eTable 1b. List of the 109 ICD-10 Codes with points awarded for each code used to calculate the Hospital Frailty Risk Score[38]

ICD Code	ICD Description	Points
F00	Dementia in Alzheimer's disease	7.1
G81	Hemiplegia	4.4
G30	Alzheimer's disease	4.0
I69	Sequelae of cerebrovascular disease	3.7
R29	Other symptoms and signs involving the nervous and musculoskeletal systems	3.6
N39	Other disorders of urinary system	3.2
F05	Delirium, not induced by alcohol and other psychoactive substances	3.2
W19	Unspecified fall	3.2
S00	Superficial injury of head	3.2
R31	Unspecified hematuria	3.0
B96	Other bacterial agents as the cause of diseases classified to other chapters	2.9
R41	Other symptoms and signs involving cognitive functions and awareness	2.7
R26	Abnormalities of gait and mobility	2.6
I67	Other cerebrovascular diseases	2.6
R56	Convulsions, not elsewhere classified	2.6
R40	Somnolence, stupor and coma	2.5
T83	Complications of genitourinary prosthetic devices, implants and grafts	2.4
S06	Intracranial injury	2.4
S42	Fracture of shoulder and upper arm	2.3
E87	Other disorders of fluid, electrolyte and acid-base balance	2.3
M25	Other joint disorders, not elsewhere classified	2.3
E86	Volume depletion	2.3
R54	Senility	2.2
Z50	Care involving use of rehabilitation procedures	2.1
F03	Unspecified dementia	2.1
W18	Other fall on same level	2.1
Z75	Problems related to medical facilities and other health care	2.0
F01	Vascular dementia	2.0
S80	Superficial injury of lower leg	2.0
L03	Cellulitis	2.0
H54	Blindness and low vision	1.9
E53	Deficiency of other B group vitamins	1.9
Z60	Problems related to social environment	1.8
G20	Parkinson's disease	1.8
R55	Syncope and collapse	1.8
S22	Fracture of rib(s), sternum and thoracic spine	1.8
K59	Other functional intestinal disorders	1.8
N17	Acute renal failure	1.8
L89	Decubitus ulcer	1.7
Z22	Carrier of infectious disease	1.7

© 2022 Sun LY et al. *JAMA Network Open.*

B95	Streptococcus/staphylococcus as cause of diseases classified to other chapters	1.7
L97	Ulcer of lower limb, not elsewhere classified	1.6
R44	Other symptoms and signs involving general sensations and perceptions	1.6
K26	Duodenal ulcer	1.6
I95	Hypotension	1.6
N19	Unspecified renal failure	1.6
A41	Other septicemia	1.6
Z87	Personal history of other diseases and conditions	1.5
J96	Respiratory failure, not elsewhere classified	1.5
X59	Exposure to unspecified factor	1.5
M19	Other arthrosis	1.5
G40	Epilepsy	1.5
M81	Osteoporosis without pathological fracture	1.4
S72	Fracture of femur	1.4
S32	Fracture of lumbar spine and pelvis	1.4
E16	Other disorders of pancreatic internal secretion	1.4
R94	Abnormal results of function studies	1.4
N18	Chronic renal failure	1.4
R33	Retention of urine	1.3
R69	Unknown and unspecified causes of morbidity	1.3
N28	Other disorders of kidney and ureter, not elsewhere classified	1.3
R32	Unspecified urinary incontinence	1.2
G31	Other degenerative diseases of nervous system, not elsewhere classified	1.2
Y95	Nosocomial condition	1.2
S09	Other and unspecified injuries of head	1.2
R45	Symptoms and signs involving emotional state	1.2
G45	Transient cerebral ischemic attacks and related syndromes	1.2
Z74	Problems related to care-provider dependency	1.1
M79	Other soft tissue disorders, not elsewhere classified	1.1
W06	Fall involving bed	1.1
S01	Open wound of head	1.1
A04	Other bacterial intestinal infections	1.1
A09	Diarrhea and gastroenteritis of presumed infectious origin	1.1
J18	Pneumonia, organism unspecified	1.1
J69	Pneumonitis due to solids and liquids	1.0
R47	Speech disturbances, not elsewhere classified	1.0
E55	Vitamin D deficiency	1.0
Z93	Artificial opening status	1.0
R02	Gangrene, not elsewhere classified	1.0
R63	Symptoms and signs concerning food and fluid intake	0.9
H91	Other hearing loss	0.9
W10	Fall on and from stairs and steps	0.9
W01	Fall on same level from slipping, tripping and stumbling	0.9
E05	Thyrotoxicosis [hyperthyroidism]	0.9

© 2022 Sun LY et al. *JAMA Network Open.*

M41	Scoliosis	0.9
R13	Dysphagia	0.8
Z99	Dependence on enabling machines and devices	0.8
U80	Agent resistant to penicillin and related antibiotics	0.8
M80	Osteoporosis with pathological fracture	0.8
K92	Other diseases of digestive system	0.8
I63	Cerebral Infarction	0.8
N20	Calculus of kidney and ureter	0.7
F10	Mental and behavioral disorders due to use of alcohol	0.7
Y84	Other medical procedures as the cause of abnormal reaction of the patient	0.7
R00	Abnormalities of heart beat	0.7
J22	Unspecified acute lower respiratory infection	0.7
Z73	Problems related to life-management difficulty	0.6
R79	Other abnormal findings of blood chemistry	0.6
Z91	Personal history of risk-factors, not elsewhere classified	0.5
S51	Open wound of forearm	0.5
F32	Depressive episode	0.5
M48	Spinal stenosis	0.5
E83	Disorders of mineral metabolism	0.4
M15	Polyarthrosis	0.4
D64	Other anemias	0.4
L08	Other local infections of skin and subcutaneous tissue	0.4
R11	Nausea and vomiting	0.3
K52	Other non-infective gastroenteritis and colitis	0.3
R50	Fever of unknown origin	0.1

© 2022 Sun LY et al. *JAMA Network Open.*

eTable 1c. Scoring rubric for the Preoperative Frailty Index

Variable	Source	Points		
		0	0.5	1
Anticholinergic risk scale	ODB	0	1–2	>2
Arrhythmia	Elixhauser	None		Present
Cancer	Elixhauser	None		Present
Cerebrovascular disease	Elixhauser	None		Present
Chronic obstructive pulmonary disease	COPD algorithm	None		Present
Dementia	Elixhauser	None		Present
Dental	ADG	None		Present
Dermatologic	ADG	None		Present
Diabetes mellitus (DM)	DM algorithm	None		Present
Dialysis	Elixhauser	None		Present
Drug or alcohol abuse	Elixhauser	None	one	Both
Heart failure (HF)	HF algorithm	None		Present
Hemiparesis	Elixhauser	None		Present
History of falls	ICD-10 code	None		Present
Home oxygen	ADP	None		Present
HOMR Score	Calculated	0–21	22–55	>55
Hypertension (HTN)	HTN algorithm	None		Present
Injury	ADG	None	minor	Major
Liver disease	Elixhauser	None		Present
Multimorbidity	Charlson score	0	1–2	>2
Myocardial Infarction (MI)	MI algorithm	None		Present
Peripheral vascular disease	Elixhauser	None		Present
Psychosocial (minor or stable)	ADG	None	minor/stable	Major
Resource use band 4–5	ADG	0–1	2–3	4–5
Rheumatic disease	Elixhauser	None		Present
Socioeconomic status	Census	Top 2 quintiles	middle quintile	Bottom 2 quintiles
Ear, nose, throat	ADG	None	stable	Unstable
Eye	ADG	None	stable	Unstable
Supported living environment	CCRS/HCD/LTC			
Weight loss	Elixhauser	None		Present

Abbreviations: ADG – Aggregated Diagnosis Group, ADP – Assistive Devices Program, CCRS – Continuing Care Reporting System, COPD – chronic obstructive pulmonary disease, HCD – Home Care Database, ICD-10, International Classification of Diseases, 10th Edition, ODB – Ontario Drug Benefits Program.

© 2022 Sun LY et al. *JAMA Network Open.*

eTable 2. Baseline Characteristics of Participants by Frailty Instrument

Variable n (%)	ACG Non-Frail	ACG Frail	Total				P Value
**ACG**	73,521 (83.1)	14,935 (16.9)	88,456	-	-	-	
**Female**	18,127 (24.7%)	4,797 (32.1%)	22,924 (25.9%)	-	-	-	<.0001
**Male**	55,394 (75.3%)	10,138 (67.9%)	65,532 (74.1%)	-	-	-	
**Age, mean (SD), yr**	65.68 (11.09)	69.21 (10.89)	66.28 (11.13)	-	-	-	<.0001
**Variable n (%)**	**HFRS 0**	**HFRS 0.1-2.0**	**HFRS 2.1-5.1**	**HFRS ≥ 5.1**	**Total**		**P Value**
**HFRS Group**	25361 (28.7)	21766 (24.6)	21836 (24.7)	19493 (22.0)	88,456	-	
**HFRS, mean (SD)**	0.00 (0.00)	1.33 (0.44)	3.37 (0.82)	9.58 (4.49)	3.27 (4.18)	-	<.0001
**Female**	5,582 (22.0)	4,997 (23.0)	5,813 (26.6)	6,532 (33.5)	22,924 (25.9)	-	<.0001
**Male**	19,779 (78.0)	16,769 (77.0)	16,023 (73.4)	12,961 (66.5)	65,532 (74.1)	-	
**Age, mean (SD), yr**	64.10 (10.91)	64.87 (10.78)	67.15 (10.82)	69.71 (11.19)	66.28 (11.13)	-	<.0001
**Variable n (%)**	**PFI 0.00-0.04**	**PFI 0.05-0.10**	**PFI 0.11-0.20**	**PFI 0.21-0.44**	**PFI 0.45-1.00**	**Total**	**P Value**
**PFI Group**	11702 (13.2)	24684 (27.9)	33111 (37.4)	18859 (21.3)	100 (0.1)	88,456	
**PFI, mean (SD)**	0.02 (0.01)	0.07 (0.02)	0.15 (0.03)	0.26 (0.05)	0.47 (0.03)	0.13 (0.09)	<.0001
**Female**	1,862 (15.9)	5,494 (22.3)	9,116 (27.5)	6,415 (34.0)	37 (37.0)	22,924 (25.9)	<.0001
**Male**	9,840 (84.1)	19,190 (77.7)	23,995 (72.5)	12,444 (66.0)	63 (63.0)	65,532 (74.1)	
**Age, mean (SD), yr**	57.96 (10.77)	64.32 (10.86)	68.03 (10.40)	70.92 (9.46)	68.67 (9.63)	66.28 (11.13)	<.0001

Abbreviations: ACG – Johns Hopkins Adjusted Clinical Groups, HFRS – hospital frailty risk score, PFI – preoperative frailty index, SD – standard deviation.

© 2022 Sun LY et al. *JAMA Network Open.*

eTable 3. **Sensitivity, Specificity, Positive and Negative Predictive Values of Each Frailty Instrument for the Estimation of PACE and Death**

eTable 3a. Sensitivity, specificity, positive and negative predictive values of each frailty instrument, for the prediction of PACE

Frailty Instrument	Sensitivity (95% CI)	Specificity (95% CI)	PPV (95% CI)	NPV (95% CI)
**ACG**	0.23 (0.22-0.23)	0.85 (0.85-0.85)	0.31 (0.30-0.32)	0.79 (0.78-0.79)
**HFRS**				
Optimal cutoff*	0.64 (0.64-0.65)	0.53 (0.53-0.53)	0.29 (0.29-0.30)	0.83 (0.83-0.84)
0.1-2.0	0.81 (0.80-0.81)	0.32 (0.31-0.32)	0.26 (0.26-0.26)	0.85 (0.84-0.85)
2.1-5.0	0.60 (0.60-0.61)	0.57 (0.57-0.58)	0.30 (0.29-0.30)	0.83 (0.83-0.83)
≥ 5.1	0.34 (0.33-0.35)	0.82 (0.81-0.82)	0.36 (0.35-0.36)	0.81 (0.80-0.81)
**PFI**				
Optimal cutoff*	0.73 (0.72-0.73)	0.45 (0.45-0.46)	0.28 (0.28-0.29)	0.85 (0.84-0.85)
0.05-0.10	0.94 (0.93-0.94)	0.15 (0.15-0.16)	0.25 (0.25-0.25)	0.89 (0.89-0.90)
0.11-0.20	0.73 (0.72-0.73)	0.45 (0.45-0.46)	0.28 (0.28-0.29)	0.85 (0.84-0.85)
0.21-0.44	0.32 (0.31-0.33)	0.82 (0.81-0.82)	0.35 (0.34-0.35)	0.80 (0.80-0.80)
0.45-1.00	0.00 (0.00-0.00)	1.00 (1.00-1.00)	0.48 (0.38-0.58)	0.77 (0.77-0.77)

* Optimal cutoff values were 1.70 for the HFRS and 0.10 for the PFI.

Abbreviations: ACG – Johns Hopkins Adjusted Clinical Groups, CI – confidence interval, PPV – positive predictive value, NPV – negative predictive value, HFRS – hospital frailty risk score, PACE – patient-defined adverse cardiovascular and noncardiovascular events, PFI – preoperative frailty index.

© 2022 Sun LY et al. *JAMA Network Open.*

eTable 3b. Sensitivity, specificity, positive and negative predictive values of each frailty instrument, for the prediction of death

Frailty Instrument	Sensitivity (95% CI)	Specificity (95% CI)	PPV (95% CI)	NPV (95% CI)
**ACG**	0.26 (0.25-0.26)	0.86 (0.86-0.86)	0.37 (0.36-0.37)	0.79 (0.78-0.79)
**HFRS**				
Optimal cutoff*	0.69 (0.89-0.70)	0.57 (0.57-0.58)	0.34 (0.33-0.34)	0.85 (0.85-0.86)
0.1-2.0	0.85 (0.84-0.85)	0.33 (0.33-0.33)	0.28 (0.28-0.29)	0.87 (0.87-0.88)
2.1-5.0	0.67 (0.67-0.68)	0.60 (0.59-0.60)	0.35 (0.34-0.35)	0.85 (0.85-0.86)
≥ 5.1	0.42 (0.41-0.42)	0.84 (0.84-0.84)	0.45 (0.45-0.46)	0.82 (0.82-0.82)
**PFI**				
Optimal cutoff*	0.68 (0.67-0.68)	0.63 (0.63-0.64)	0.37 (0.36-0.37)	0.86 (0.86-0.86)
0.05-0.10	0.96 (0.96-0.96)	0.16 (0.16-0.16)	0.27 (0.26-0.27)	0.93 (0.92-0.93)
0.11-0.20	0.80 (0.79-0.80)	0.48 (0.47-0.48)	0.33 (0.32-0.33)	0.88 (0.88-0.89)
0.21-0.44	0.42 (0.42-0.43)	0.85 (0.85-0.85)	0.47 (0.47-0.48)	0.82 (0.82-0.83)
0.45-1.00	0.00 (0.00-0.00)	1.00 (1.00-1.00)	0.74 (0.64-0.82)	0.76 (0.76-0.76)

* Optimal cutoff values were 1.80 for the HFRS and 0.13 for the PFI.

Abbreviations: ACG – Johns Hopkins Adjusted Clinical Groups, CI – confidence interval, PPV – positive predictive value, NPV – negative predictive value, HFRS – hospital frailty risk score, PFI – preoperative frailty index.

© 2022 Sun LY et al. *JAMA Network Open.*

eTable 4. Differences in AUROC Between Frailty Instruments

Comparator	Time (days)	Unadjusted AUROC Difference (95% CI) for PACE	Unadjusted AUROC Difference (95% CI) for Death
HFRS-ACG	30	0.16 (0.14-0.18)	0.18 (0.16-0.19)
	90	0.14 (0.12-0.16)	0.19 (0.17-0.21)
	365	0.13 (0.11-0.15)	0.18 (0.15-0.20)
	730	0.12 (0.10-0.14)	0.16 (0.14-0.18)
	1825	0.12 (0.10-0.13)	0.14 (0.12-0.16)
	3650	0.10 (0.09-0.12)	0.12 (0.11-0.14)
PFI-ACG	30	0.13 (0.11-0.15)	0.15 (0.13-0.17)
	90	0.11 (0.09-0.12)	0.15 (0.13-0.17)
	365	0.10 (0.08-0.12)	0.16 (0.15-0.18)
	730	0.10 (0.08-0.12)	0.19 (0.17-0.20)
	1825	0.12 (0.11-0.14)	0.16 (0.15-0.18)
	3650	0.16 (0.15-0.17)	0.19 (0.17-0.20)
PFI-HFRS	30	-0.03 (-0.04- -0.02)	-0.03 (-0.04- -0.02)
	90	-0.04 (-0.04- -0.03)	-0.04 (-0.05- -0.03)
	365	-0.03 (-0.04- -0.02)	-0.02 (-0.03- -0.01)
	730	-0.02 (-0.02- -0.01)	-0.01 (-0.01- 0.00)
	1825	0.01 (0.00-0.01)	0.02 (0.02-0.03)
	3650	0.06 (0.005-0.07)	0.06 (0.06-007)

Abbreviations: ACG – Johns Hopkins Adjusted Clinical Groups, CI – confidence interval, HR – hazard ratio, HFRS – hospital frailty risk score, PACE – patient-defined adverse cardiovascular and noncardiovascular events, PFI – preoperative frailty index, AUROC – area under the receiver-operating characteristic curve.

© 2022 Sun LY et al. *JAMA Network Open.*

eTable 5. Adjusted Hazard Ratios of PACE by Frailty Instrument

Frailty Instrument	Model Adjusting for Demographic variables[1] HR (95% CI)	P-Value	Model Adjusting for Demographics & Comorbidities[2] HR (95% CI)	P-Value
**ACG**	1.42 (1.38-1.47)	< 0.0001	1.25 (1.21-1.29)	< 0.0001
**HFRS**				
per 1 unit	1.08 (1.08-1.09)	< 0.0001	1.06 (1.06-1.07)	< 0.0001
0.1-2.0	1.24 (1.19-1.29)	< 0.0001	1.19 (1.14-1.25)	< 0.0001
2.1-5.0	1.62 (1.56-1.69)	< 0.0001	1.49 (1.43-1.56)	< 0.0001
≥ 5.1	2.67 (2.56-2.78)	< 0.0001	2.14 (2.05-2.24)	< 0.0001
**PFI**				
per 0.1 unit	1.58 (1.55-1.61)	< 0.0001	1.43 (1.40-1.48)	< 0.0001
0.05-0.10	1.49 (1.39-1.59)	< 0.0001	1.38 (1.30-1.48)	< 0.0001
0.11-0.20	2.14 (2.01-2.28)	< 0.0001	1.80 (1.69-1.92)	< 0.0001
0.21-0.44	3.39 (3.17-3.61)	0.0001	2.45 (2.27-2.64)	< 0.0001
0.45-1.00	7.23 (5.41-9.67)	< 0.0001	3.48 (2.59-4.69)	0.345

[1] **Demographic variables include:** age, sex, rural residence, income quintile, and urgency of surgery.

[2] **Comorbidities include:** remote myocardial infraction (MI), recent MI, history of percutaneous coronary intervention, hypertension, atrial fibrillation, left ventricular ejection fractions, heart failure, cerebrovascular disease, peripheral arterial disease, chronic obstructive pulmonary disease or asthma, diabetes, morbid obesity, hypothyroidism, anemia, dialysis, renal disease, livers disease, primary malignancy, metastatic malignancy, and dementia.

Abbreviations: ACG – Johns Hopkins Adjusted Clinical Groups, CI – confidence interval, HR – hazard ratio, HFRS – hospital frailty risk score, PACE – patient-defined adverse cardiovascular and noncardiovascular events, PFI – preoperative frailty index.

eTable 6. Adjusted Hazard Ratios of Death by Frailty Instrument

Frailty Instrument	Model Adjusting for Demographic variables[1] HR (95% CI)	P-Value	Model Adjusting for Demographics & Comorbidities[2] HR (95% CI)	P-Value
**ACG**	1.54 (1.49-1.59)	< 0.0001	1.23 (1.19-1.27)	< 0.0001
**HFRS**				
per 1 unit	1.09 (1.09-1.09)	< 0.0001	1.05 (1.05-1.06)	< 0.0001
0.1-2.0	1.25 (1.19-1.31)	< 0.0001	1.17 (1.11-1.23)	< 0.0001
2.1-5.0	1.79 (1.72-1.88)	< 0.0001	1.49 (1.43-1.56)	< 0.0001
≥ 5.1	3.39 (3.26-3.54)	< 0.0001	2.12 (2.03-2.22)	0.017
**PFI**				
per 0.1 unit	1.96 (1.93-1.99)	< 0.0001	1.39 (1.6-1.43)	< 0.0001
0.05-0.10	1.61 (1.49-1.74)	< 0.0001	1.38 (1.287-1.49)	< 0.0001
0.11-0.20	2.63 (2.44-2.83)	< 0.0001	1.81 (1.681.96)	< 0.0001
0.21-0.44	5.65 (5.24-6.09)	< 0.0001	2.46 (2.26-2.68)	0.002
0.45-1.00	14.54 (11.45-18.48)	0.821	2.81 (2.19-3.61)	0.037

[1] **Demographic variables include:** age, sex, rural residence, income quintile, and urgency of surgery.

[2] **Comorbidities include:** remote myocardial infraction (MI), recent MI, history of percutaneous coronary intervention, hypertension, atrial fibrillation, left ventricular ejection fractions, heart failure, cerebrovascular disease, peripheral arterial disease, chronic obstructive pulmonary disease or asthma, diabetes, morbid obesity, hypothyroidism, anemia, dialysis, renal disease, livers disease, primary malignancy, metastatic malignancy, and dementia.

Abbreviations: ACG – Johns Hopkins Adjusted Clinical Groups, CI – confidence interval, HR – hazard ratio, HFRS – hospital frailty risk score, PACE – patient-defined adverse cardiovascular and noncardiovascular events, PFI – preoperative frailty index.

© 2022 Sun LY et al. *JAMA Network Open.*

eFigure 1. Patient Flow Diagram

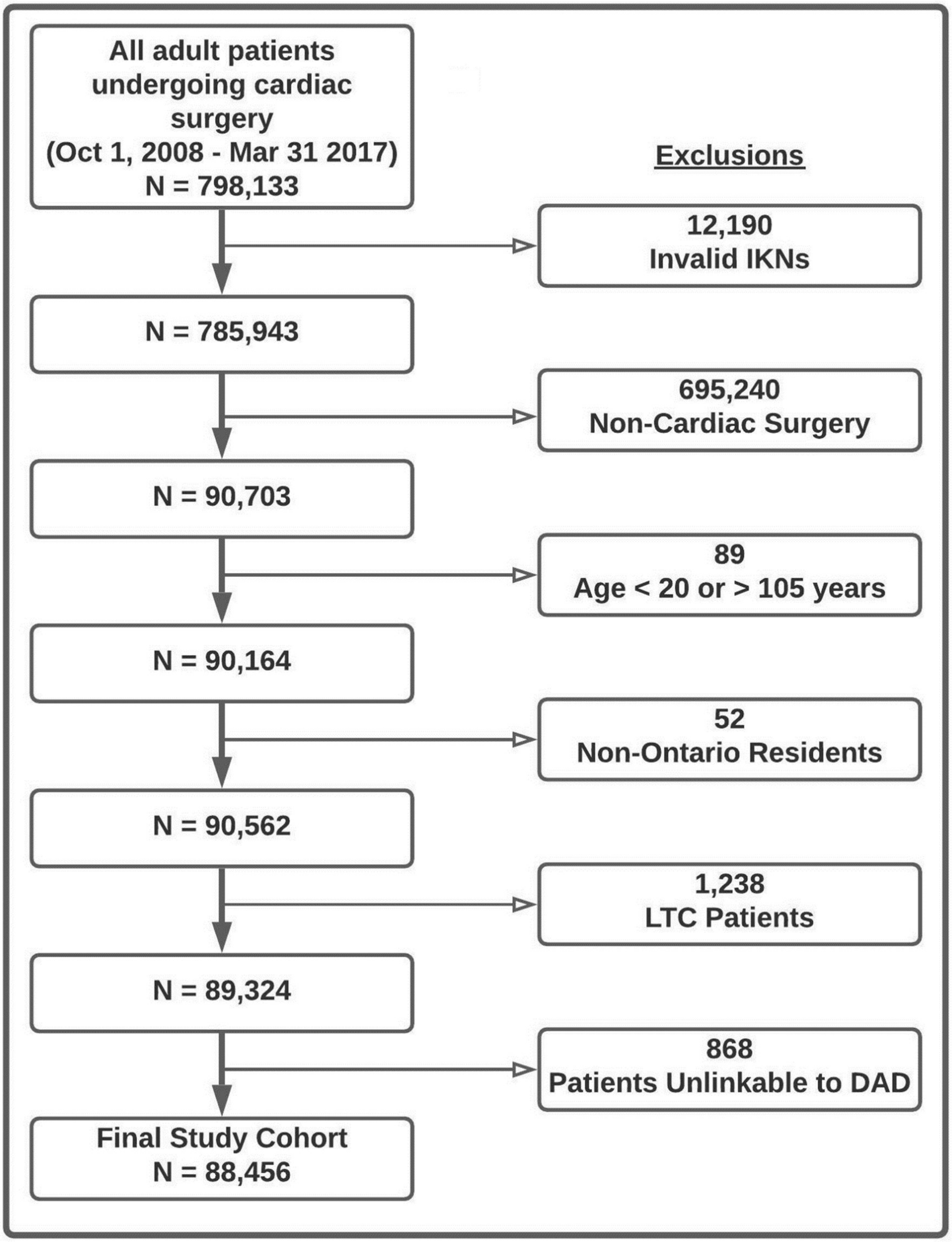

Abbreviations: AD – Discharge Abstract Database, IKN – ICES Key Number, LTC – long-term care.

© 2022 Sun LY et al. *JAMA Network Open.*

eFigure 2. Unadjusted Hazard Ratios and Unadjusted Receiver Operating Characteristic Curves for Individual PACE Events and Each Frailty Instrument

eFigure 2a. Unadjusted hazard ratios of the association between individual PACE events and each of the frailty instruments

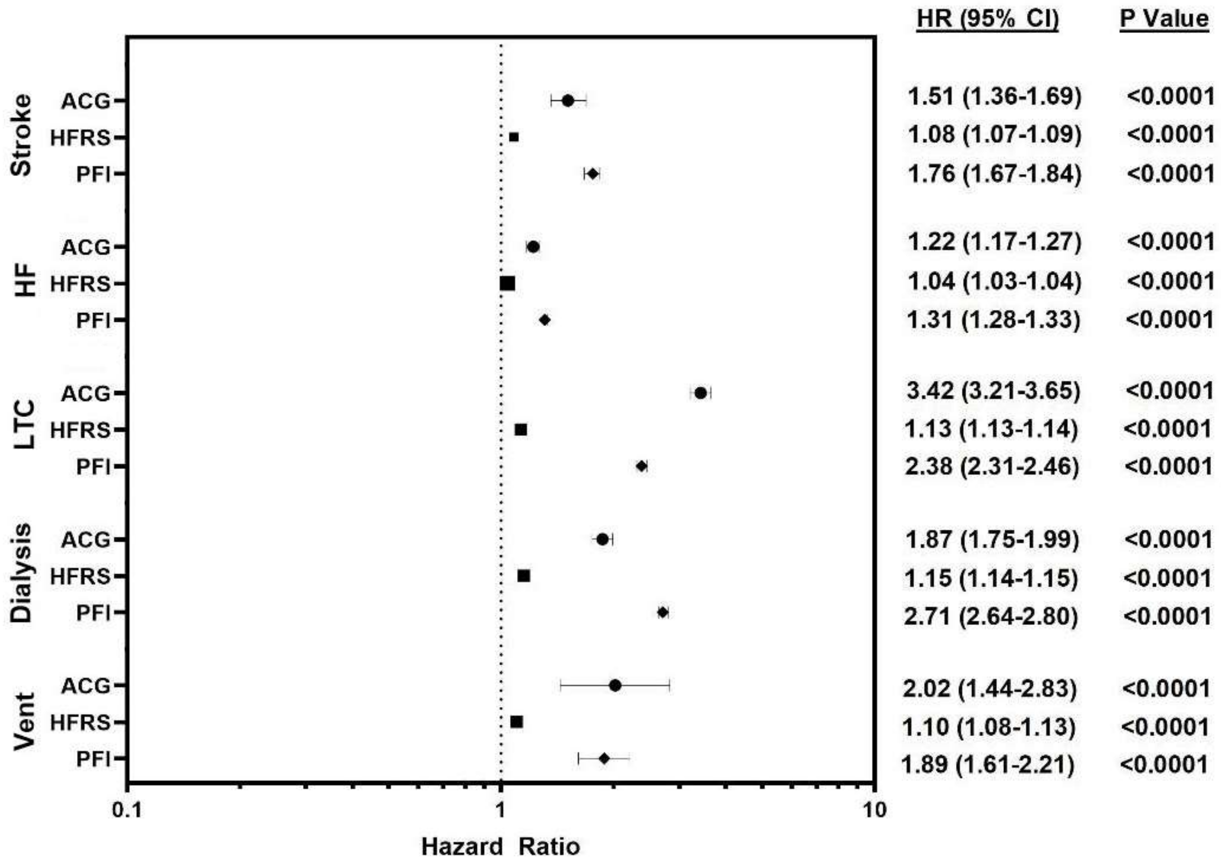

* Hazard ratios are provided per 1 unit increment for HFRS and per 0.1 unit increment for PFI.

Abbreviations: ACG – Johns Hopkins Adjusted Clinical Groups, CI – confidence interval, HF –heart failure, HFRS – hospital frailty risk score, LTC – long term care admission, PACE – patient-defined adverse cardiovascular and noncardiovascular events, PFI – preoperative frailty index, Vent – ventilator dependence.

© 2022 Sun LY et al. *JAMA Network Open.*

eFigure 2b. Unadjusted receiver-operating characteristic curves for the prediction of individual PACE events according to each of the frailty instruments

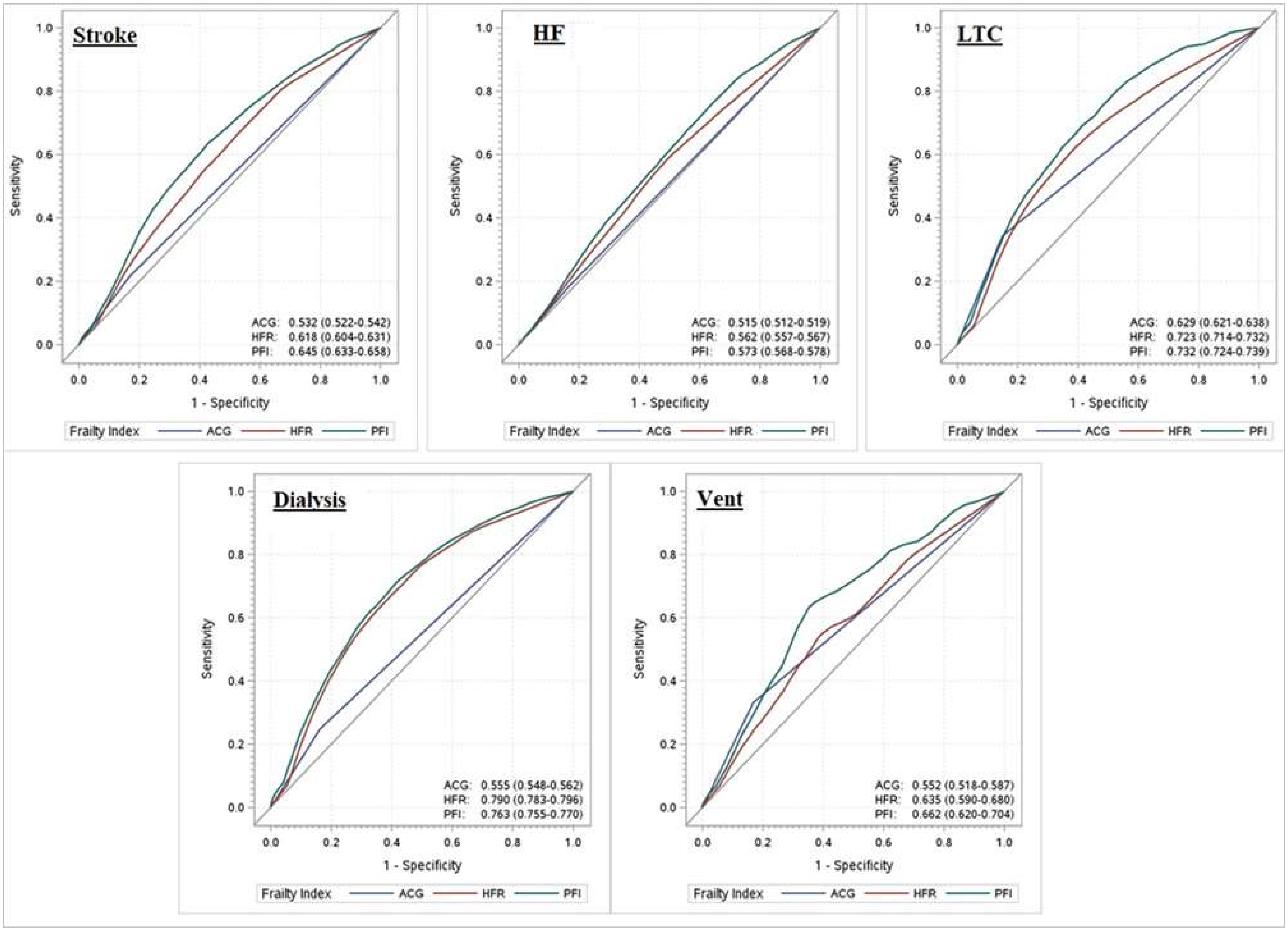

Abbreviations: ACG – Johns Hopkins Adjusted Clinical Groups, HF –heart failure, HFR – hospital frailty risk score, LTC – long term care admission, PACE – patient-defined adverse cardiovascular and noncardiovascular events, PFI – preoperative frailty index, Vent – ventilator dependence.

© 2022 Sun LY et al. *JAMA Network Open.*

eFigure 3. Time-Dependent Receiver Operating Characteristic Curves of the Frailty Instruments for the Estimation of PACE

eFigure 3a. Time-dependent receiver-operating characteristic curves of the frailty instruments for the prediction of PACE, adjusted for age, sex, socioeconomic class and procedure urgency

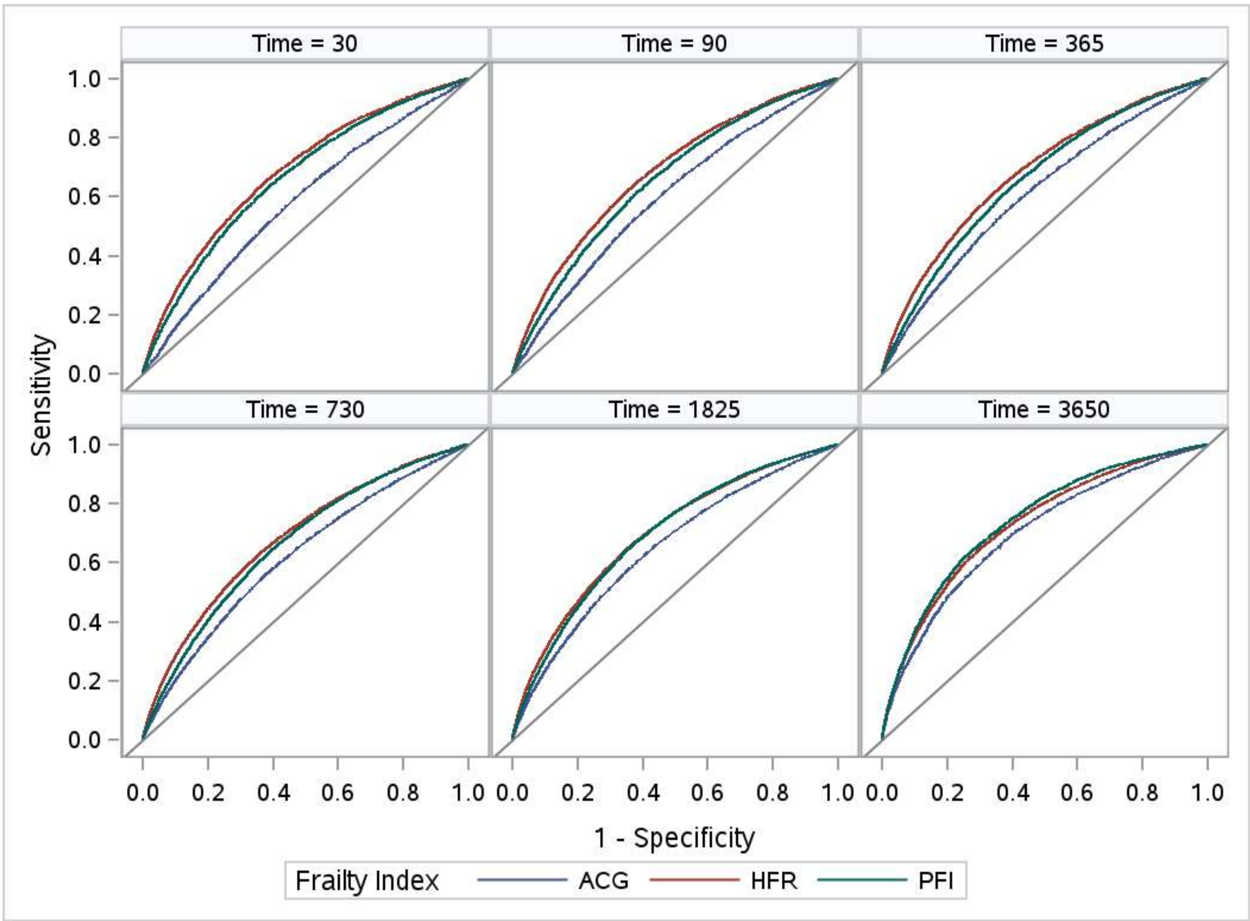

© 2022 Sun LY et al. *JAMA Network Open.*

eFigure 3b. Time-dependent receiver-operating characteristic curves of the frailty instruments for the prediction of PACE, adjusted for age, sex, socioeconomic class, procedure urgency and patient comorbidities

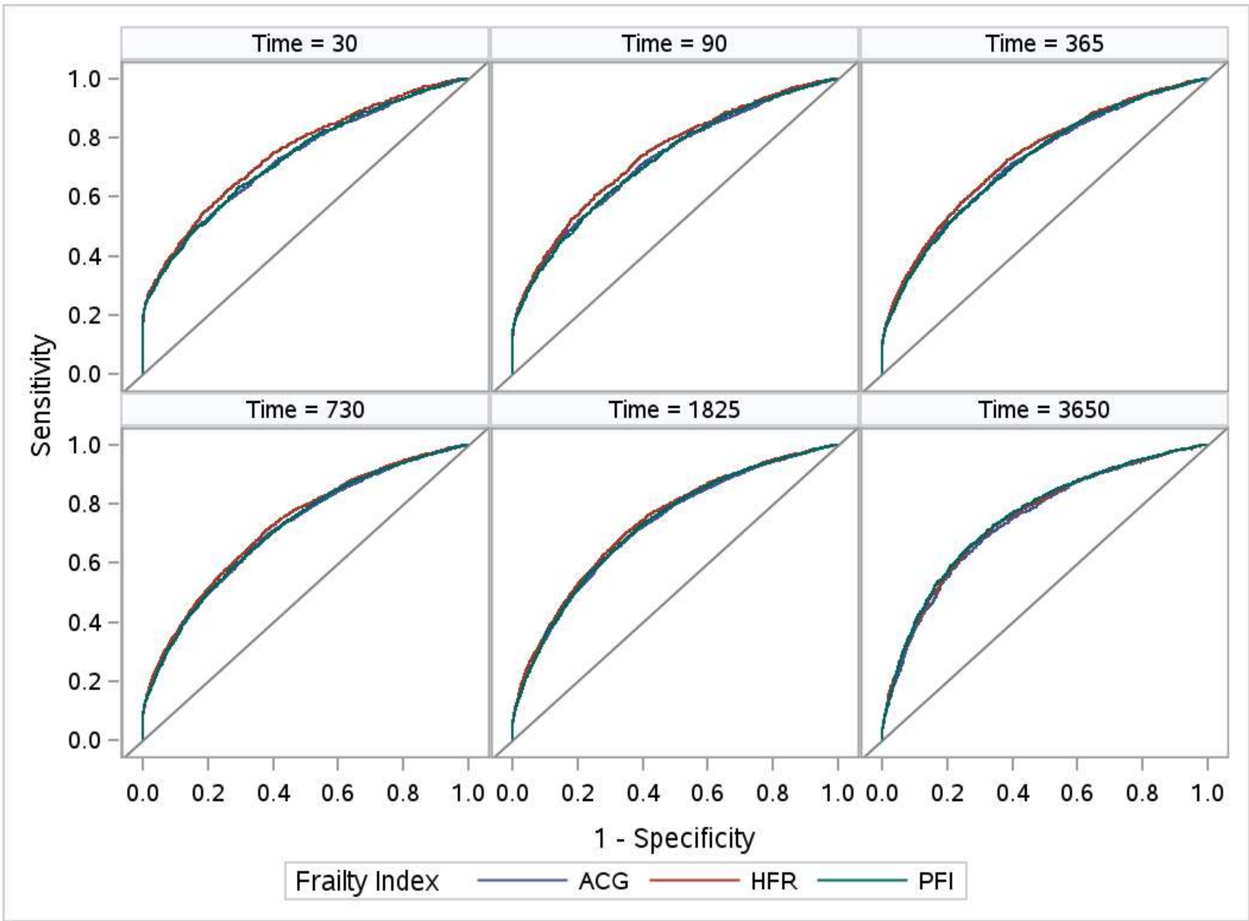

© 2022 Sun LY et al. *JAMA Network Open.*

eFigure 4. Unadjusted Estimated Long-term Survival by Each Frailty Index

eFigure 4a. Unadjusted estimated long-term survival by Johns Hopkins ACG frailty status. The shaded areas represent 95% confidence intervals.

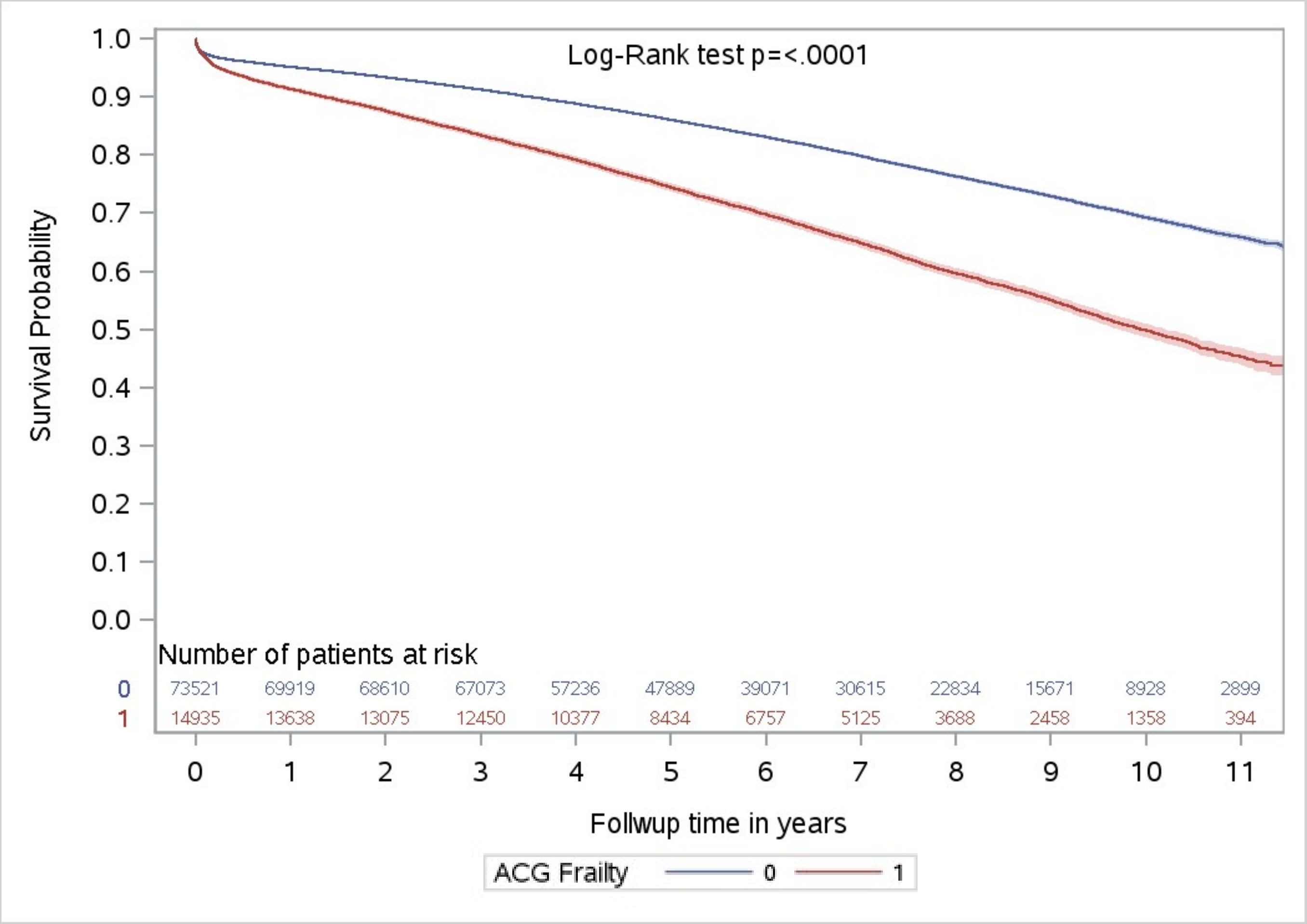

Abbreviations: ACG – Johns Hopkins Adjusted Clinical Groups.

© 2022 Sun LY et al. *JAMA Network Open.*

eFigure 4b. Unadjusted estimated long-term survival by Hospital Frailty Risk Score. The shaded areas represent 95% confidence intervals.

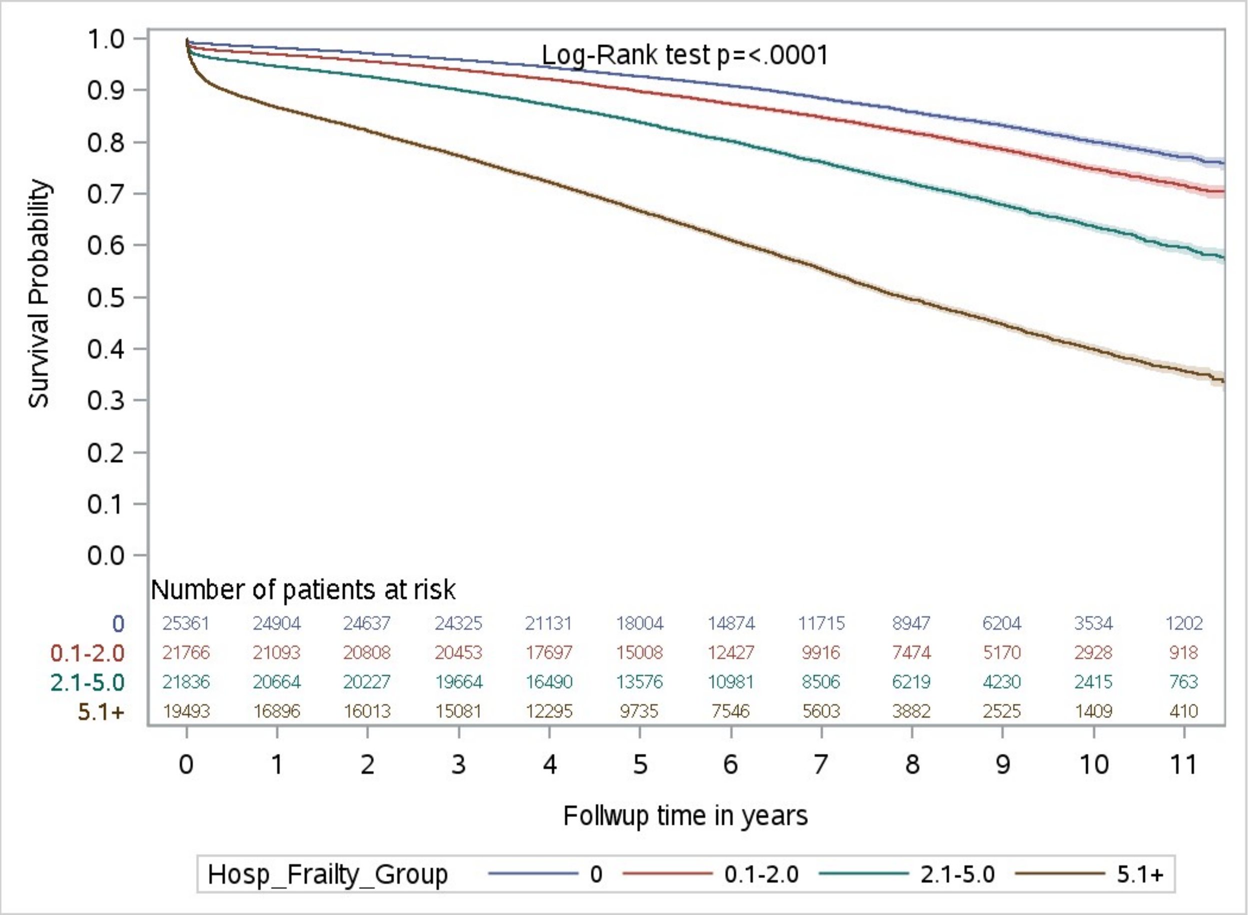

Abbreviations: Hosp_Frailty_Group – hospital frailty risk score**.**

© 2022 Sun LY et al. *JAMA Network Open.*

eFigure 4c. Unadjusted estimated long-term survival by Preoperative Frailty Index. The shaded areas represent 95% confidence intervals.

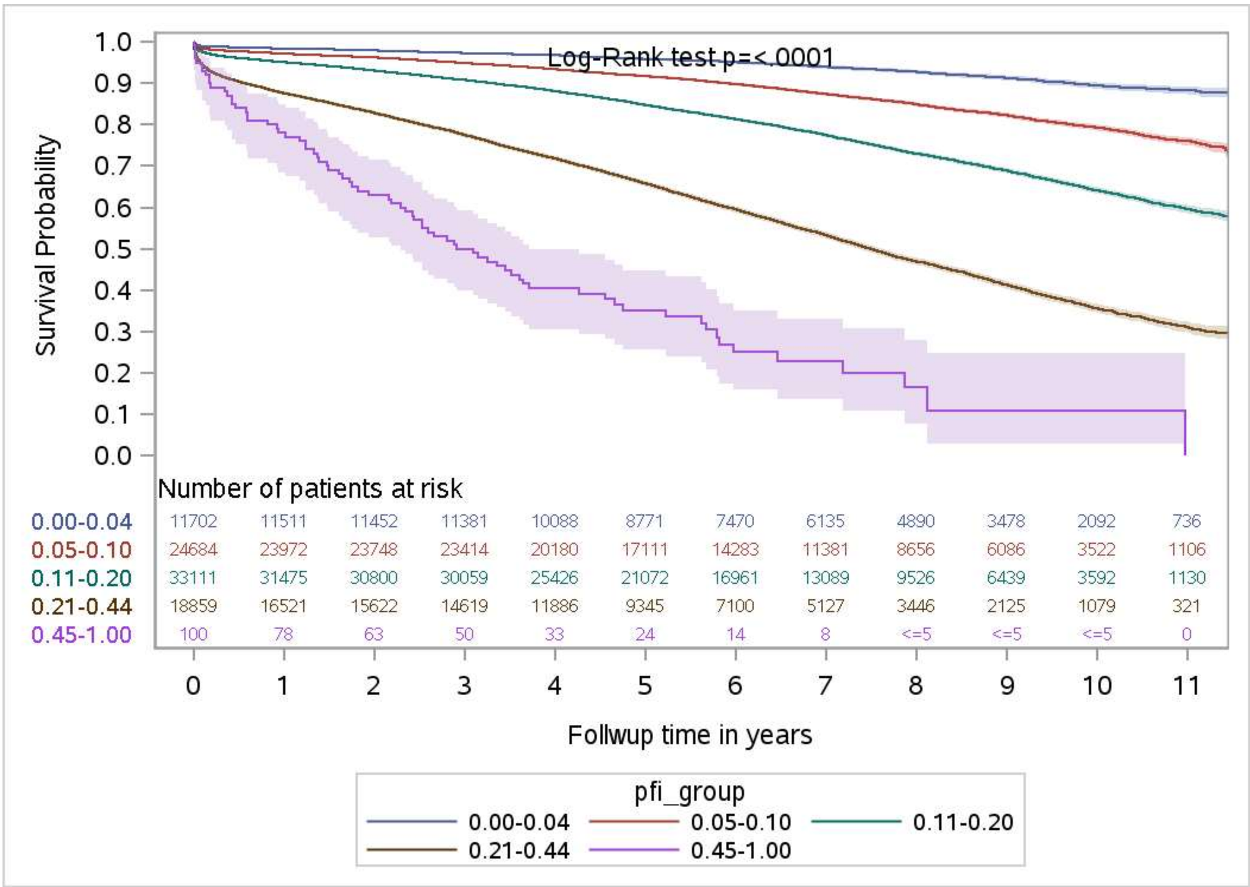

Abbreviations: PFI – preoperative frailty index.

© 2022 Sun LY et al. *JAMA Network Open.*

eFigure 5. Unadjusted Hazard Ratios of the Association Between Death and Each Frailty Instrument

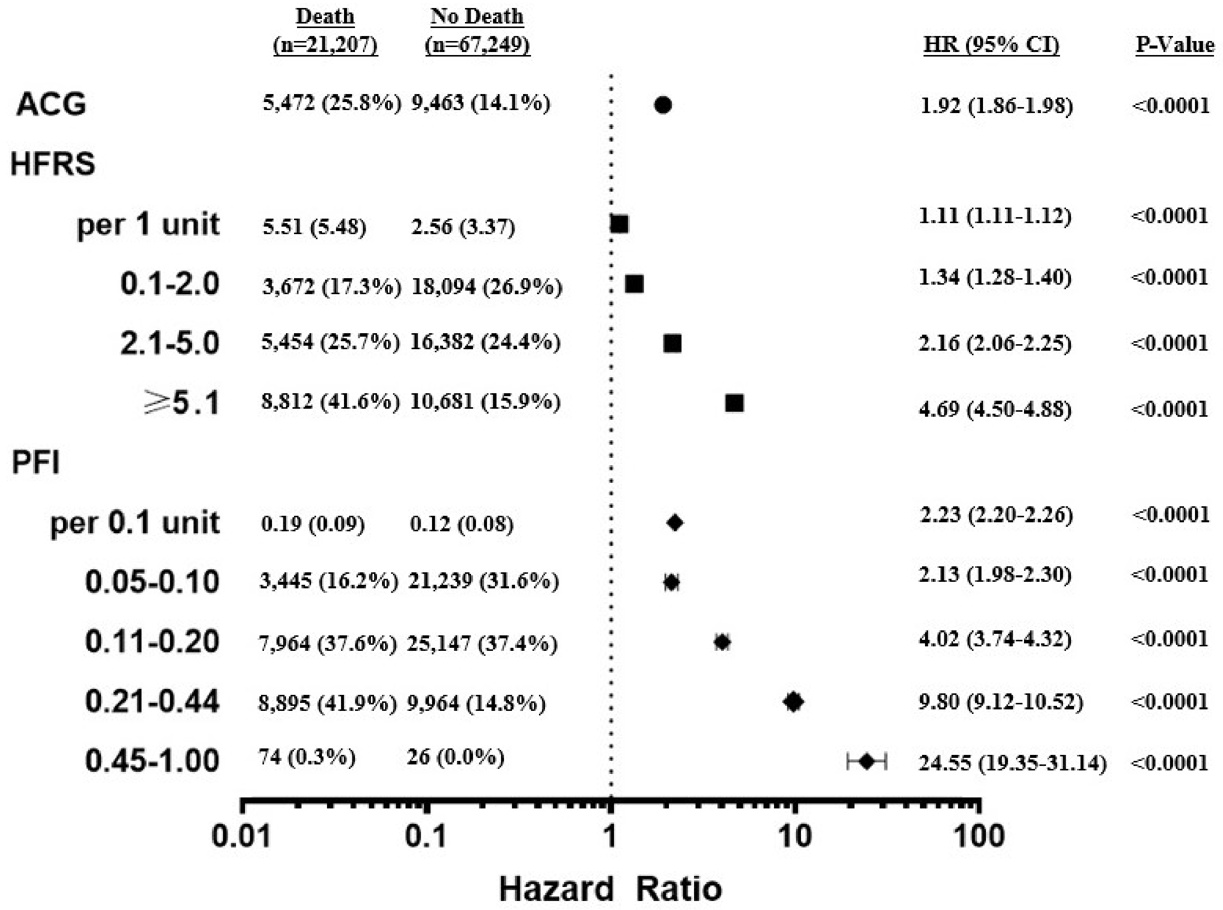

Abbreviations: ACG – Johns Hopkins Adjusted Clinical Groups, CI – confidence interval, HFRS – hospital frailty risk score, PACE – patient-defined adverse cardiovascular and noncardiovascular events, PFI – preoperative frailty index.

© 2022 Sun LY et al. *JAMA Network Open.*

eFigure 6. Unadjusted Time-Dependent Receiver Operating Characteristic Curves and Plots of Areas Under the Receiver Operating Characteristic Curve (AUROC) of the Frailty Instruments for the Estimation of Death

eFigure 6a. Unadjusted time-dependent receiver-operating characteristic curves of the frailty instruments for the prediction of death

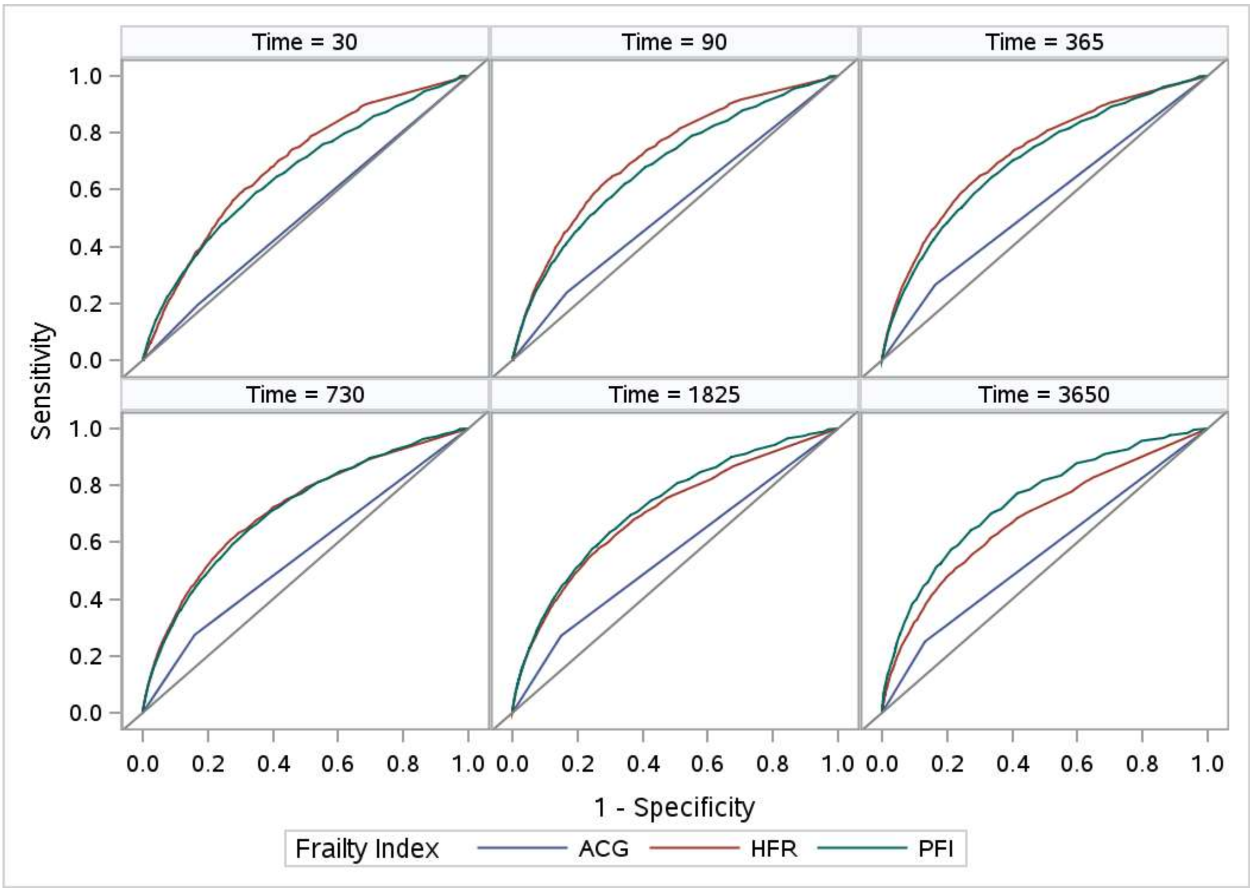

Abbreviations: ACG – Johns Hopkins Adjusted Clinical Groups, HFRS – hospital frailty risk score, PFI – preoperative frailty index.

© 2022 Sun LY et al. *JAMA Network Open.*

eFigure 6b. Plot of areas under the receiver-operating characteristic curves (AUROC) of the frailty instruments over time, for the prediction of death. The shaded areas represent 95% confidence intervals of the AUROC values.

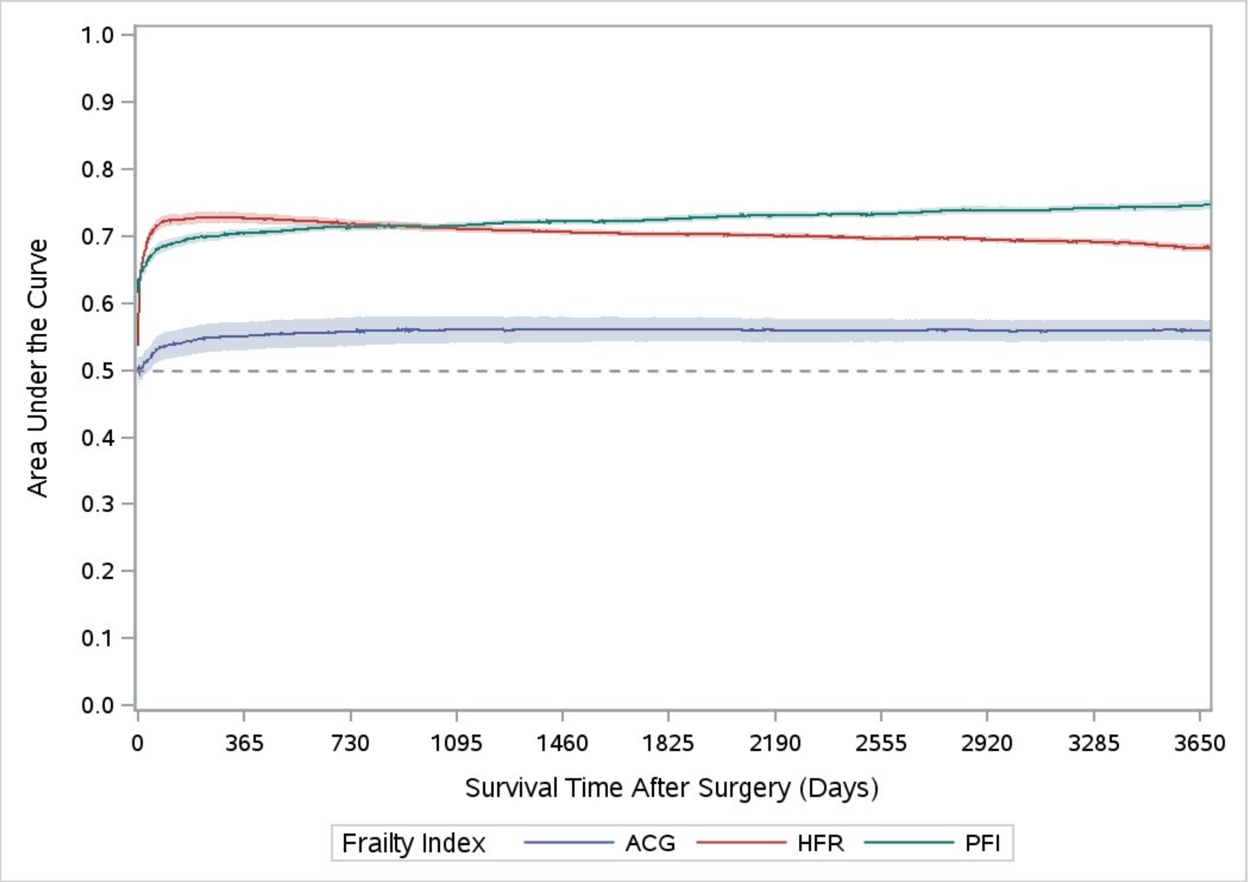

Abbreviations: ACG – Johns Hopkins Adjusted Clinical Groups, HFRS – hospital frailty risk score, PFI – preoperative frailty index.

© 2022 Sun LY et al. *JAMA Network Open.*

eFigure 7. Time-Dependent Receiver Operating Characteristic Curves of the Frailty Instruments for the Estimation of Death

eFigure 7a. Time-dependent receiver-operating characteristic curves of the frailty instruments for the prediction of death, adjusted for age, sex, socioeconomic class and procedure urgency

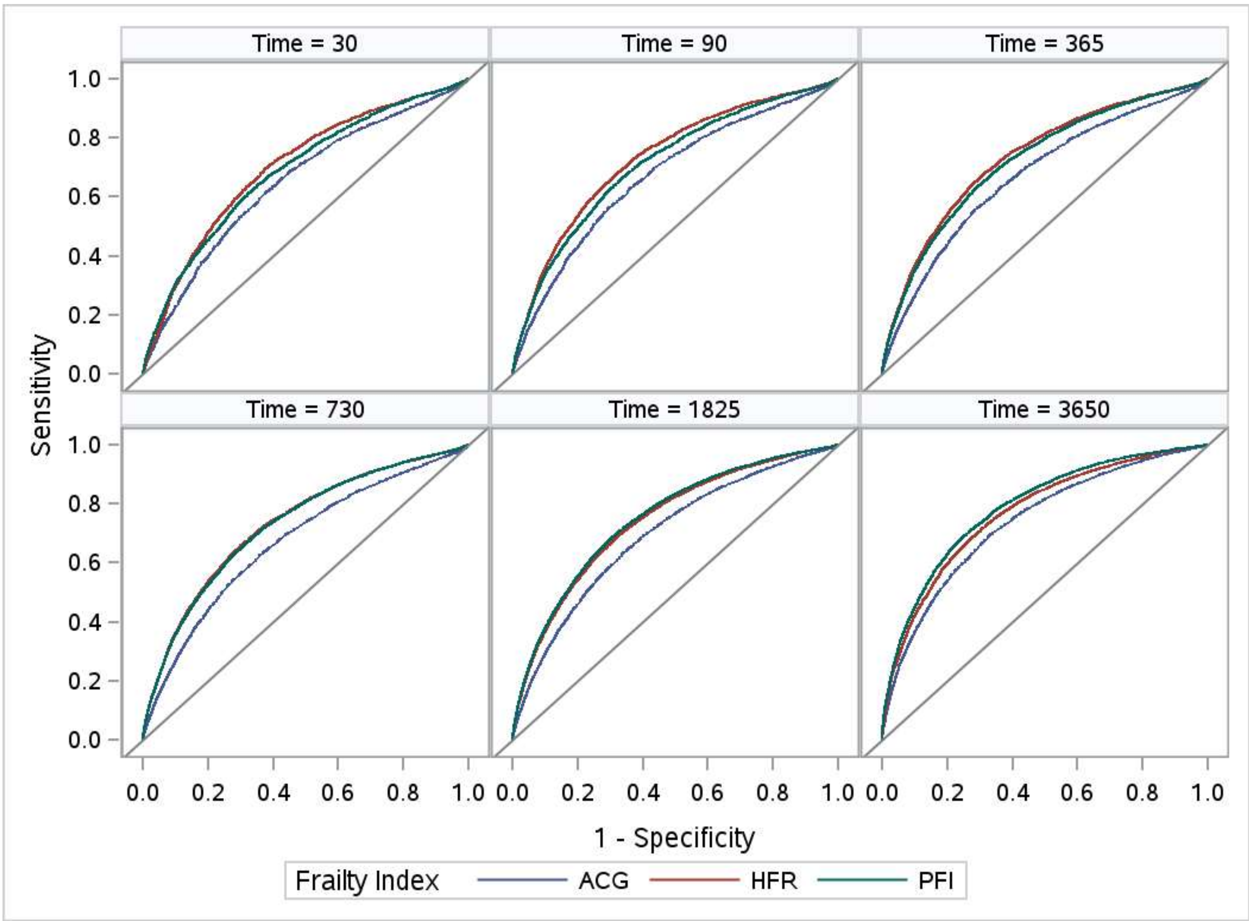

© 2022 Sun LY et al. *JAMA Network Open.*

eFigure 7b. Time-dependent receiver-operating characteristic curves of the frailty instruments for the prediction of death, adjusted for age, sex, socioeconomic class, procedure urgency and patient comorbidities

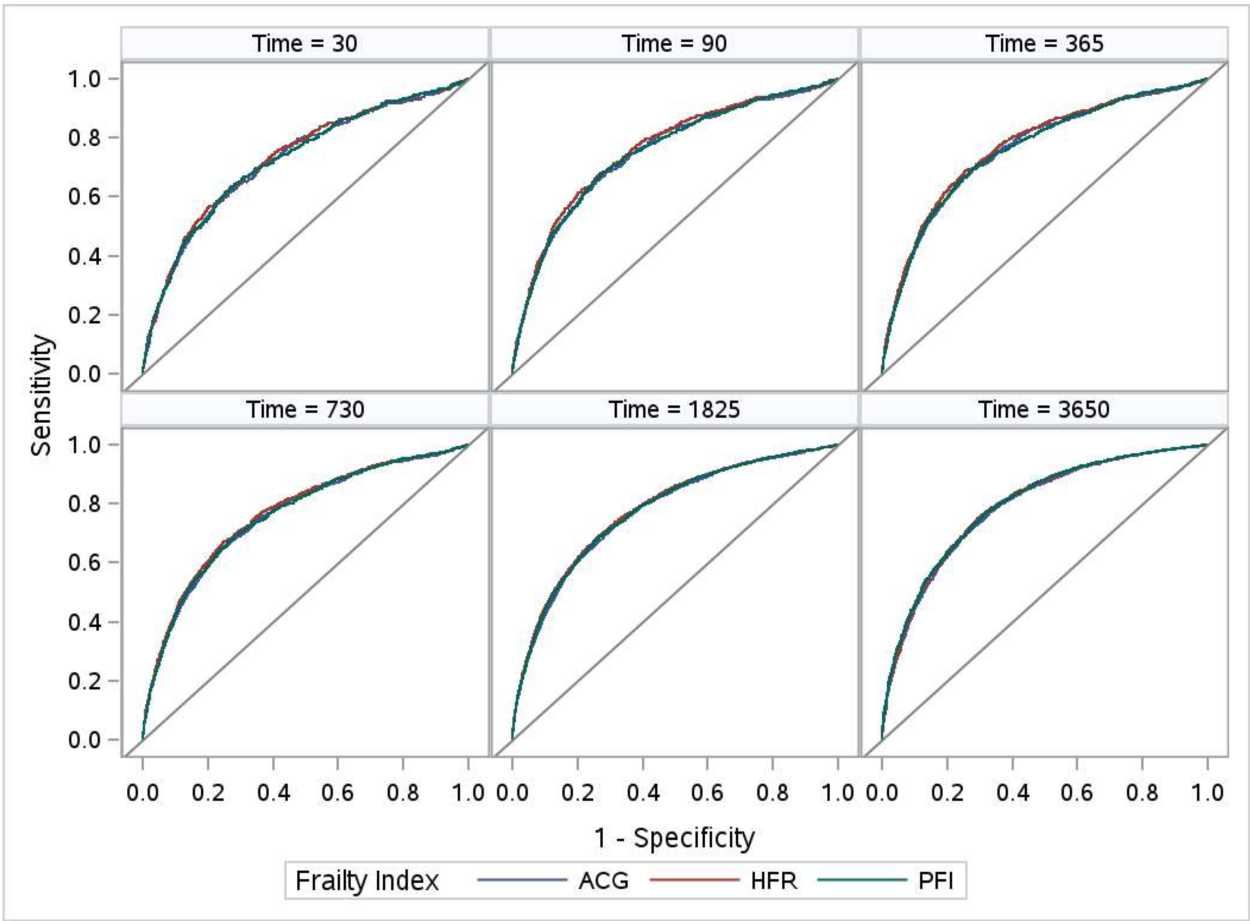

© 2022 Sun LY et al. *JAMA Network Open.*
